# Early stages of sympatric homoploid hybrid speciation in crater lake cichlid fishes

**DOI:** 10.1038/s41467-022-33319-4

**Published:** 2022-10-06

**Authors:** Melisa Olave, Alexander Nater, Andreas F. Kautt, Axel Meyer

**Affiliations:** 1 Department of Biology, University of Konstanz, 78457 Konstanz, Germany; 2 Present Address: Argentine Dryland Research Institute of the National Scientific and Technical Research Council (IADIZA-CONICET), 5500 Mendoza, Argentina; 3 Present Address: Interfaculty Bioinformatics Unit, University of Bern, 3012 Bern, Switzerland; 4 Present Address: Department of Organismic and Evolutionary Biology, Harvard University, Cambridge, MA USA

**Keywords:** Adaptive radiation, Evolutionary genetics, Conservation genomics, Genetic hybridization

## Abstract

Homoploid hybrid speciation (i.e., hybrid speciation without a change in ploidy) has traditionally been considered to be rare in animals. Only few accepted empirical examples of homoploid hybrid speciation in nature exist, and in only one previous case (insects) was it convincingly shown that this process occurred in complete sympatry. Here, we report an instance of sympatric homoploid hybrid speciation in Midas cichlid fishes in Crater Lake Xiloá, Nicaragua. The hybrid lineage, albeit at an early stage of speciation, has genomically and phenotypically diverged from both of its two parental species. Together with a distinct stable isotope signature this suggests that this hybrid lineages occupies a different trophic niche compared to the other sympatric Midas cichlid species in Crater Lake Xiloá.

## Abstract (web summary)

Here, the authors present a rare example of sympatric homoploid hybrid speciation, without ploidy changes, in the Midas cichlid fishes from Nicaragua. Midas cichlid hybrids occupy a different ecological niche, likely facilitated by body shape adaptations.

## Introduction

Classically considered an evolutionary dead-end, hybridization has recently been recognized to have played a widespread and important role in driving speciation, including in the formation of adaptive radiations^1–6^. Hybridization can drive the formation of a stable hybrid zone^7^, reverse speciation^8^, reinforce speciation^9^, or even generate a new separately evolving lineage through hybrid speciation^10^. The importance of hybrid speciation has been debated for decades^2,3,10,11,12^ and the theoretical obstacles remain, given that an incipient hybrid species has to maintain high fitness while becoming reproductively isolated from both parental species. Hybrid speciation is more common in plants than animals, likely due to their higher rate of producing viable polyploid offspring^13^, a process that sometimes generates reproductive isolation with both parental species in a single generation^2,3^. In contrast, only a few examples of homoploid (i.e., without changes in ploidy) hybrid speciation in nature are known, especially when strictly adhering to the three criteria proposed by Schumer et al.^11^ (but see refs. 12 and 14) to determine the strength of evidence for homoploid hybrid speciation. These criteria include (i) reproductive isolation has been established, (ii) the new species has arisen due to hybridization detectable in the genome, and (iii) reproductive isolating mechanisms were directly derived from hybridization. Nonetheless, a few cases of homoploid hybrid speciation have been reported (at least providing evidence for criteria (i) and (ii)), but their reproductive isolation relies on geographic separation to both parental species (e.g., bats^15^, swordtail fishes^16,17,18^) or at least to one parental species (*Heliconius*^19^, sparrows^20^, and Darwin finches^21^). Yet, for tephritid fruit flies it has been demonstrated that homoploid hybrid speciation in animals is possible, even if hybrids coexist in sympatry with both parental species^22^. The hybrid lineage avoids competition with its parental species through a host shift, i.e., it feeds and mates on a different flowering plant than either parental species. Thus, adaptation to separate microenvironments can potentially facilitate overcoming both obstacles^23^: allowing reproductive isolation and avoiding a decrease in fitness due to competition with the parental species.

Previous work on the Midas cichlid species complex (*Amphilophus citrinellus* spp.) from Nicaragua showed that this is an extraordinarily recent radiation in which sympatric speciation has been convincingly demonstrated in several crater lakes^24,25,26^. A key ecological trait with a putative role in their diversification is body shape, exemplified by the often bimodal distribution of elongated (“limnetic”) versus deeper-bodied (“benthic”) fish^27^ within a lake. Distinct body shapes allow for differences in swimming performance, thus enabling fish to exploit different niches available across a lake: more elongated bodies are associated with a more piscivorous diet, while deeper-bodied fish feed predominantly on algae and benthic invertebrates^27,28^. A founder population from the older Great Lake Managua colonized Crater Lake Xiloá (CL Xiloá) only about 4300 years ago^26^. CL Xiloá is a small crater lake (only ~2 km in diameter) harboring at least four endemic Midas cichlid species which have speciated in sympatry: *Amphilophus amarillo, A. viridis, A. sagittae,* and *A. xiloaensis*^24,25,29^. Genetic differentiation is low among the sympatric Midas cichlid species in CL Xiloá, and hybridization has been previously suspected between *A. sagittae* (limnetic species) and *A. xiloaensis* (benthic species)^26,30^.

Here, we show that these hybrids are in the early stages of hybrid speciation occurring in complete sympatry with both parental species. Hybrids are morphologically distinct and occupy a different trophic niche from the other four sympatric species in CL Xiloá including both parental species.

## Results

### Genomic evidence of hybridization in Midas cichlids from CL Xiloá

This study is based on sequenced genomes of 120 individuals (mean coverage: 26×)^26^ (Source data file 1, Supplementary Fig. 1) of the focal lineages *A. sagittae* ((*n* = 13), *A. xiloaensis* (*n* = 10), putative hybrids (*n* = 11), and backcrosses (21), as well as the other two endemic Midas cichlid species found in CL Xiloá, *A. amarillo* (*n* = 21), and *A. viridis* (*n* = 19), and the outgroup *A. citrinellus* from Great Lake Managua (*n* = 25).

**Fig. 1:**
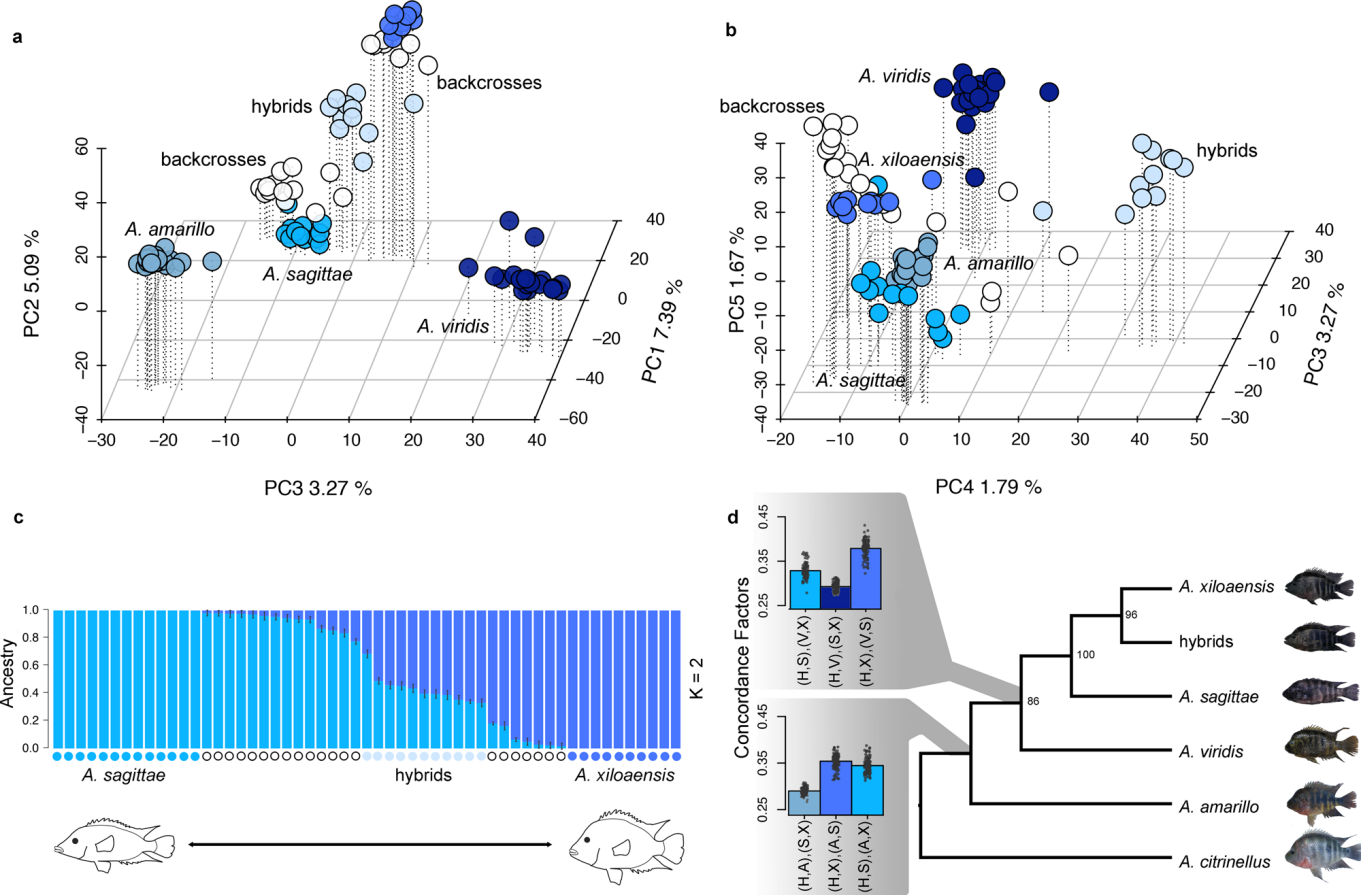
Genomic evidence of hybridization. **a**, **b** PCA constructed based on 175,942 SNPs (sampled every 5 kb along the genome). “White” symbols correspond to individuals that are not classified either as parental or hybrids (later on identified as backcrosses, see Fig. 2b). **c** Admixture plot including error bars constructed on 175,942 SNPs error bars. **d** Species tree reconstructed with SVDquartets based on 88,369 SNPs. Bootstrap support (200 pseudoreplicates) is shown on the nodes. Concordance factors of species quartets of interest (split of *A. sagittae*, *A. xiloaensis,* and hybrids) are shown on the nodes (*n* = 100 individual quartets per species quartet; see all possible quartet comparisons in Supplementary Fig. 5, abbreviations S = *A. sagittae*, X = *A. xiloaensis*, H = hybrids, A = *A. amarillo*, V = *A. viridis*). Source data are provided as Source data file 1.

To illustrate genetic clustering, we used a Principal Component Analysis (PCA) based on 175,942 single nucleotide polymorphisms (SNPs) (Fig. 1a, b, Supplementary Figs. 2 and 3). The first three PC axes capture genetic variation separating the four described species, with PC2 and PC3, in particular, visualizing the distinctiveness of individuals resulting from genetic admixture between *A. sagittae* and *A. xiloaensis*. These individuals are genetically intermediate between both species (light blue circles, from now on “hybrids”) and are recovered as sister clade to *A. xiloaensis* in a neighbor-joining tree (Supplementary Fig. 4) and a coalescent-based phylogenetic analysis (Fig. 1d). Concordance factor calculations (CFs; proportion of SNPs supporting the split in a particular 4-taxon group [or quartet]^29^) show extensive genome-wide discordance among all Midas cichlid species in CL Xiloá (Supplementary Fig. 5), an expected pattern due to the very recent divergence of this clade. Yet, it is expected that splits between sister taxa are recovered with higher CFs, while CFs for the two remaining splits should be approximately equal in the absence of post-divergence gene flow^31^. Interestingly, the split between hybrids and *A. xiloaensis* exhibits a higher CF, but there is a deviation in the remaining two CFs in favor of the hybrids and *A. sagittae* (Fig. 1d; Supplementary Fig. 5). Moreover, a hybrid detection test (HyDe) was highly significant (*γ* = 0.62; *p*-value = 0.006, Z score = 2.54; Supplementary Table 1), providing further support that the intermediate position in the PCA of these individuals is a signal of hybridization between the two parental species. The hybrid individuals exhibit a similar degree of genetic differentiation from either parental species but are slightly less differentiated from *A. xiloaensis* (median F_st_ *A. xiloaensis* vs. hybrids = 0.0275; median F_st_ *A. sagittae* vs. hybrids = 0.0282; Supplementary Fig. 6). These F_st_ values are smaller compared to those between the two parental species (median F_st_ *A. sagittae* vs. *A. xiloaensis* = 0.0633, Supplementary Fig. 6). Some individuals of admixed ancestry are more difficult to classify as either “parental species” or “hybrids” (shown in white in Fig. 1a, b; later identified as backcrosses, see below). Furthermore, while an admixture analysis detects mixed ancestry between *A. sagittae* and *A. xiloaensis* for several individuals when restricting the analysis to two clusters (Fig. 1c, *K* = 2, CV = 0.148), the same individuals are assigned to a distinct group when allowing for a third cluster (Supplementary Fig. 7; *K* = 3, CV = 0.1558). Consistent with the support for a separate hybrid cluster, PC4 clearly separates the hybrids from all other Midas cichlid species in the lake (Fig. 1b, Supplementary Figs. 2 and 3). Taken together, these results suggest that a distinct genetic composition of the hybrid group might not be simply the result of contemporary hybridization or backcrossing and prompted us to conduct further analyses to address this hypothesis specifically.

### Early stages of a separately evolving hybrid lineage in CL Xiloá

**Fig. 2:**
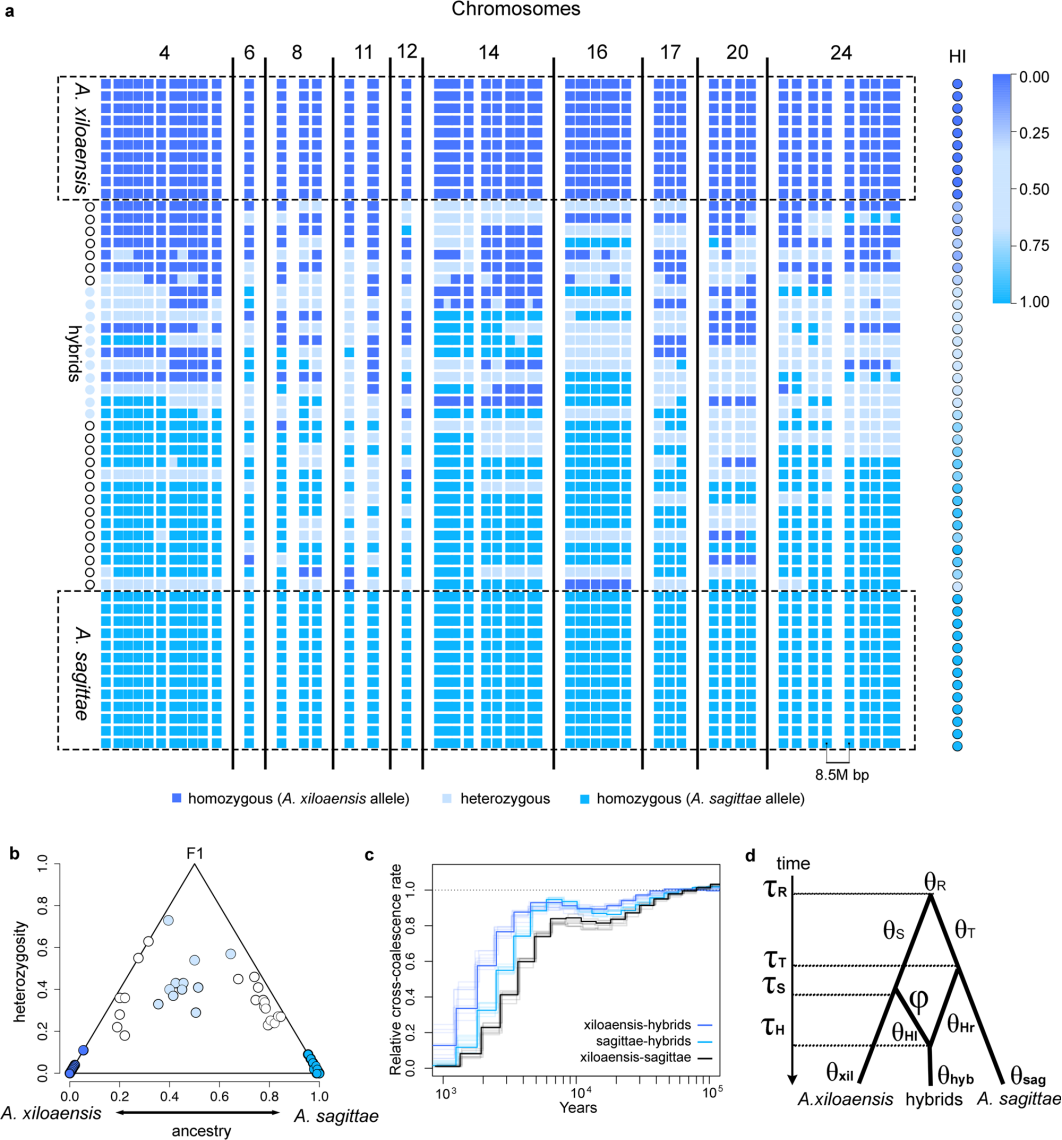
Early stages of a separately evolving lineage with hybrid origin. **a** Matrix comparing hybrid genotypes at 52 SNPs fixed between the parental species (see also Supplementary Fig. 9 for a relaxed approach under 95% fixation rate and Supplementary Figs. 11–13 and Supplementary Table 9 for ancestry tract comparisons along the whole genomes). Separation of SNP columns is in log-scale according to their physical distance within each chromosome (scale shown in chromosome 24, bottom). On the right, hybrid index estimations obtained based on these 52 SNPs is shown in color code according to the scale bar (see Supplementary Table 6 for inbreeding coefficient calculated along the whole genome). **b** HIest result showing the range of possible hybrid genomic proportions in terms of ancestry (S) and interclass heterozygosity (H). Color code for individuals is identical to the PCA in Figs. 1a, b and 2a. **c** Changes in relative cross-coalescence rate (RCCR) through time estimated with MSMC. Light lines are estimations obtained for each of the 10 bootstrap replicates. **d** Demographic coalescent-based model evaluated using the MSci BPP program. Estimations include the number of generations (*τ*), population size (*θ*), and the genetic contribution (*φ*) parameters. Source data are provided as Source data files 1 and 2.

To further explore the putative hybrid origin scenario, we assessed other measures of genetic differentiation between the hybrid individuals and the two parental species. First, we investigated whether any private high-frequency alleles have accumulated in the hybrid lineage, that is, a biallelic SNP that is only polymorphic in the hybrid lineage (minimum frequency of private allele > =0.5) and completely monomorphic in the other four Midas cichlid species in CL Xiloá. We found a total of 38 such SNPs with private high-frequency alleles. Notably, these SNPs are located in or close to 15 different annotated genes along eight chromosomes (Supplementary Tables 2 and 3, all SNPs with private alleles display read coverage as well as variant quality metrics that are indistinguishable from the rest of the genome, Supplementary Fig. 1). These genes are associated with different gene ontology (GO) terms for biological processes (Supplementary Fig. 8, Supplementary Table 4). Only eight out of the 38 alleles are found exclusively in hybrids (or backcrosses) when comparing them against a larger dataset including all Midas cichlid species (333 whole genomes representing 22 species/populations from nine lakes^26^, Supplementary Table 5). This suggests that at least 30 out of the 38 alleles are most likely derived from standing genetic variation. In addition, when looking at private alleles in other species in the lake, twenty were found in *A. xiloaensis* and two in the other parental species *A. sagittae*, 24 in *A. viridis*, and 187 in the oldest endemic lineage of this crater lake radiation, *A. amarillo*. Sample sizes did not significantly affect private allele counts (*p*-value = 0.2015, F_*df=3*_ = 2.659), thus we report absolute values. Next, we searched for SNPs fixed between the two parental species and found a total of 52 SNPs (Supplementary Table 7; here, no minimum physical distance threshold was applied among SNPs). These 52 SNPs correspond to 18 annotated genes and a gene ontology (GO) analysis found several different biological processes associated with them (Supplementary Table 7 and Supplementary Fig. 8). Based on these fixed (and diagnostic) markers, we constructed a matrix to explore the level of genomic recombination in the full spectrum of hybrid individuals (11 hybrids plus 21 backcrosses, Fig. 2a). It is possible that these 32 hybrids represent contemporary admixed individuals during ongoing sympatric divergence of *A. sagittae* and *A. xiloaensis*. In other words, these hybrids might simply be F1s, unlikely to persist in time as a separately evolving lineage. Any F1 hybrid individual is expected to display only heterozygote genotypes at these loci^32^. However, of the 32 sampled individuals none appeared to be an F1 hybrid (Fig. 2a). Instead, we detected evidence for recombination of parental haplotypes even among physically close SNPs (<1000 bp; Fig. 2a and Supplementary Fig. 9; Supplementary Table 7). Next, we used the Hlest program^32^, an explicit method for hybrid estimation designed to detect parental individuals, backcrosses, F1s, and older hybrid generations (≥F2) when considering ancestry (S) and heterozygosity (H) at diagnostic markers. Parental individuals are expected to fall into opposite corners at the bottom of the triangle plot (in Fig. 2b; H = 0, S = either 0 or 1). F1 individuals are expected to cluster at the top of the triangle (H = 1 and S = 0.5), while backcrosses are expected to approach the triangle lines. Isolated hybrid individuals (≥F2) are expected to move down in the triangle over time, due to a reduction in heterozygosity in each generation, while maintaining an approximately intermediate ancestry value of S = 0.5. We implemented HIest (Fig. 2b) using a matrix of 148 SNPs with a minimum allele frequency ≥95% in each parental species (here, we only included SNPs with a minimum distance of 50 kb to reduce the probability of linkage). We find no support for any F1 hybrid individuals in our sample (Fig. 2b). Instead, individuals (Fig. 2b — indicated in light blue) that exhibit approximately intermediate ancestry (S) to both parental species have an increased but, importantly, not complete (i.e., none individual H = 1) heterozygosity (in addition, see inbreeding coefficient calculated using the whole genomes in Supplementary Table 6 and Supplementary Fig. 10). This lower heterozygosity compared to expected levels for F1s is consistent with predominantly exclusive reproduction among members of the hybrid lineage for several generations (≥F2). In addition, this analysis supports the presence of backcrosses, represented with white symbols in Fig. 2b (also shown in white in Fig. 1a–c). Estimates of ancestry tracts along the whole genomes of hybrids and backcrosses show extensive evidence of mixed ancestry and recombination (Supplementary Fig. 11). Genomes of hybrids exhibit intermediate ancestry tract lengths from *A. xiloaensis* and *A. sagittae*, with slightly greater (but not statistically significant) tract lengths from *A. xiloaensis*, while backcrosses carry significantly greater ancestry tract lengths from their respectively pure parental species (Supplementary Figs. 12 and 13; Supplementary Table 9).

Having found evidence suggesting the presence of an evolutionary distinct hybrid lineage, we next aimed to infer the timing and population dynamics of its isolation. We applied the Multiple Sequentially Markovian coalescent (MSMC) method^33^ to estimate changes in effective population size (N_e_) through time (Supplementary Fig. 14) and compared the relative cross-coalescence rate (RCCR) as a proxy of temporal gene flow dynamics between parental species and hybrids (Fig. 2c). A RCCR value equal to one is expected under panmixia, while zero is expected under complete isolation. Our results show that RCCR between parental species drops substantially about 5000 years ago, indicating the beginning of divergence between parental species shortly after the hypothesized colonization of CL Xiloá^30,26^. Shortly thereafter the same pattern is shown in the pairwise comparisons between each parental species and hybrids. For all comparisons, RCCR reaches its minimum value at about 1000 years ago indicating near complete genetic isolation. Isolation of hybrids from *A. sagittae* seems to be stronger and happened earlier compared to reproductive isolation from *A. xiloaensis*. In contrast, backcrosses do not reach RCCR = 0 with any of the two parental species (Supplementary Fig. 14). Finally, we estimated the number of generations that the hybrid lineage seems to have been evolving separately from the parental species using the coalescent-based program Msci^34^ (Fig. 2d). The hybrid lineage is characterized by approximately equal contributions from each parental species (*φ* = 0.546; 95% HPD = 0.505–0.587). While the divergence between parental species is dated at 2578 generations ago (95% HPD = 2369–2794; Table 1), the hybrid lineage is estimated to have originated only 242 generation ago (95% HPD = 221–264, approximate generation time = 1.5 years). While this result indicates a younger divergence of hybrids compared to our MSMC analysis, the difference might be due to model assumptions: Msci does not account for continuous gene flow between lineages. Thus, Msci could be underestimating the true age of hybrid origin, given that reproductive isolation to the parental species is likely establishing gradually. Despite this difference in time estimation, we note that, importantly, in both cases isolation is detected in the young hybrids.

**Table 1:** Demographic model

Parameter		Mean	95% HPD interval
R	*N*_e_	44,301	42,589–46,029
	*τ*	2578	2368–2794
S	*N*_e_	390	183–692
T	*N*_e_	230	142–371
H	*N*_e_	875	102–2412
	*τ*	251	231−272
Hr	*N*_e_	139	91–212
	*τ*	252	231–272
*A. sagittae*	*N*_e_	23,280	8926–46,072
*A. xiloaensis*	*N*_e_	17,329	14,179–20,797
Hybrids	*N*_e_	6369	1210–14,286
	*τ*	242	221–264
*φ*		0.5461	0.505–0.587

Results of demographic analysis using MSci for parameter estimations in correspondence with Fig. 2d.

*N*_e_ effective population size, *τ* generations, *HPD* highest posterior density.

### Ecological and morphological differentiation of hybrids

**Fig. 3:**
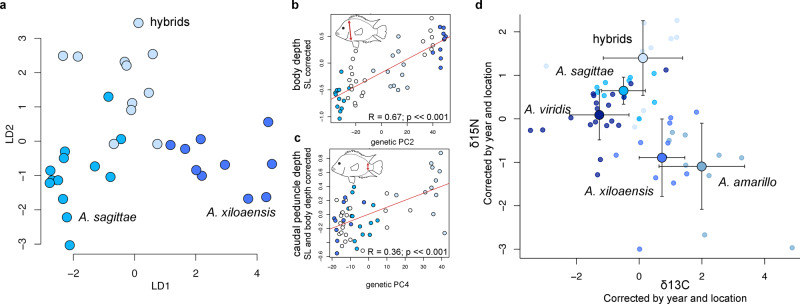
Ecological and morphological differentiation of hybrids. **a** Scatter plot of LD1 and LD2 scores obtained from a linear discriminant function based on geometric morphometric data. **b** Scatter plots and linear model fits between body depth and genetic PC2. Statistical test: F = 108.5 on 1 and 53 DF, *p*-value 1.967 × 10^−14^***. **c** Scatter plots and linear model fits between caudal peduncle depth (landmarks 6–12) and genetic PC4. Statistical test: F = 29.5 on 1 and 53 DF, *p*-value = 1.431 × 10^−06^***. **d** Scatter plot of means and standard deviations of δ^13^C isotope and δ^15^N isotope signatures in muscle tissue of individuals from all Midas cichlid species in Crater Lake Xiloá, from biologically independent hybrids *n* = 9, *A. sagittae* *n* = 10, *A. xiloaensis* *n* = 11, *A. viridis* *n* = 19 and *A. amarillo* *n* = 10. Source data are provided as Source data file 1.

Using geometric morphometric data obtained after digitization of 27 landmarks (LM), semi-landmarks, and helper points on photographs of the same individuals used for the genomic analyses (Supplementary Fig. 15), we explored the level of morphological differentiation between hybrids and both parental species. Hybrids are similar in standard length compared to each parental species (Supplementary Fig. 16, Supplementary Table 10) and have an intermediate body depth, eye width, and head angle compared to both parental species (Supplementary Fig. 16). Interestingly, the caudal peduncle depth is significantly greater in hybrids than parental species and backcrosses, as well as in any other Midas cichlid species in CL Xiloá (Supplementary Fig. 16; Supplementary Table 11). The caudal peduncle is the region connecting the caudal fin and the vertebrae column, and its shape affects the power that the fish transmits to the caudal fin and the speed at which it can swim^35^. A discriminant analysis based on body shape revealed three well-separated and morphologically distinct groups corresponding to the two parental species and the hybrids (Fig. 3a; Supplementary Fig. 17). Specifically, LD1 is highly correlated with body depth (Supplementary Fig. 18) and separates the two parental species. Body depth is a key adaptive trait that correlates with the genetic differentiation of parental species along genetic PC2 (*R*^2^ = 0.67, *p* << 0.001; Fig. 3b). Interestingly, LD2 captures relevant information corresponding to the novel body shape that distinguishes hybrids from both parental species: differences in caudal peduncle depth (Supplementary Fig. 18). This trait is correlated with genetic PC4 (*R*^2^ = 0.36, *p* << 0.001; Fig. 3c; Supplementary Fig. 19).

In order to obtain a more direct measure of ecological niche differentiation, we also analyzed stable isotope signatures of all Midas cichlid species inhabiting CL Xiloá. Significant statistical differences are detected among species (MANCOVA *p*-value << 0.001, F_*df=4*_ = 41.3994). Benthic ecomorphs (*A. xiloaensis* and *A. amarillo*) exhibit an increased level of δ^13^C isotope and a decreased level of δ^15^N with respect to *A. sagittae* and *A. viridis* (Fig. 3d), concordant with patterns previously observed in other Midas cichlid species^36,37^. The hybrid individuals display a different pattern from both parental species, as well as the other two species in CL Xiloá, with an intermediate δ^13^C isotope concentration, and an increased δ^15^N isotope concentration that likely indicates a shift to a higher trophic level than either parental species (Fig. 3d).

## Discussion

Several recent empirical studies have conducted genomic analyses to examine the role of hybridization during the formation of adaptive radiations, especially in the form of providing genetic raw material that can be re-shuffled (e.g., refs. 5, 6, 21, 38, 39, 40). However, studying hybrid speciation has been historically challenging, only rarely could hybrid speciation be documented to have contributed to speciation, much less the formation of adaptive radiations^4^, possibly due to a lack of data and methodological limitations^29,41^. The small and young cichlid fish radiation that arose in sympatry in a young crater lake (∼4300 years old) of less than two kilometers in diameter provides an ideal system in which to investigate whether hybridization might have contributed to rapid sympatric speciation^36^. Our results identified the early stages of a rare case of homoploid hybrid speciation in sympatry. Although backcrosses seem to be common (white symbols in Figs. 1a, b and 2b), we have found strong genomic evidence (Figs. 1a, b and 2a–d; Table 1; Supplementary Figs. 2–4, 7, 9, 12–14; Supplementary Tables 2, 5, 9) and differences in morphology (Fig. 3c; Supplementary Figs. 16, 17, 19, Supplementary Table 11) demonstrating that this hybrid lineage is evolving separately from its parental species and backcrosses. The evidence for this new species includes the presence of a number of high-frequency alleles in the species of hybrid origin not present in other Midas cichlid species in CL Xiloá (Supplementary Tables 2–4, Supplementary Fig. 8). Furthermore, in terms of ecological niche evolution and trophic specialization this new lineage seems to occupy the highest trophic level of all Midas cichlid species in CL Xiloá (Fig. 3d). Increased δ^15^N isotope signatures in Midas cichlid fishes have been associated with limnetic ecomorphs and with a more piscivore rather than planktivore diet^36,37,42^. Previously, a lower proportion of mollusks was found in the guts of the limnetic parental species *A. sagittae*, compared to the other benthic species in CL Xiloá (including *A. xiloaensis*)^27^. It is possible that the increased δ^15^N isotope signatures in hybrids indicate an increased predatory, piscivore feeding behavior compared to other sympatric Midas cichlid species. Conquering a novel trophic niche might have been a key aspect during the evolution of this lineage since it leads to a reduction in competition with both parental species, as well as other sympatric Midas species in CL Xiloá. Differences in body shape might have contributed to such a niche shift (Fig. 3a), since these changes likely affect swimming performance^27,26^—a trait that is likely to affect the ability to catch evasive prey higher up in the trophic chain such as fish (as indicated by the SIA data). Previous studies on the Midas cichlid radiation have focused on comparing body depth as an important trait associated with divergent selection (e.g., refs. 26, 27, 30, 36, 42, 43), but caudal peduncle depth has not received as much attention. Hybrids display an intermediate body depth compared to both parental species and a deeper caudal peduncle than any other species in CL Xiloá, including backcrosses (Supplementary Fig. 16 and Supplementary Table 11). It seems plausible that differences in caudal peduncle depth play an important role in driving the divergence of the hybrid-origin lineage from the two parental species (Fig. 3c). Moreover, given the higher trophic level occupied by the new species (Fig. 3d), it might suggest that this hybrid-origin lineage indeed became a better piscivore predator possibly caused by the surmised body-shape-driven improved swimming performance.

Reproductive isolation during (non-polyploid) sympatric speciation is known to develop gradually^44,26^ and this seems to apply as well to this young hybrid lineage. Based on the presence of backcrosses, postzygotic isolation mechanisms (again in support of the hypothesis that trophic specialization is paramount) might be suggested to play a more important role in maintaining the separateness of the hybrid lineage than prezygotic isolation. However, reproductive isolation in cichlid fishes might depend on the combination of multiple reproductive barriers (reviewed in ref. 45).

Despite the presence of backcrosses in nature, based on genomic, morphological, and trophic niche analyses we provide evidence that hybridization is driving a distinct hybrid lineage in the early stages of speciation. This work raises the question of how often hybrid speciation has resulted in new species in cichlid fishes more generally. In the Midas cichlid radiation, given that most species of the *Amphilophus cintrinellus* species complex radiation originated in sympatry and remain interfertile to date (as evidenced by ongoing gene flow)^26,29^ more examples might be found in the future. Furthermore, given the exceedingly rapid speciation rates of Midas cichlids^26^, we are afforded the unique opportunity to study the evolution of this hybrid-origin species during our lifetime. In conclusion, here we have identified a rare case of incipient sympatric homoploid hybrid speciation — to the best of our knowledge the only example of this type of speciation in vertebrates to date.

## Methods

### Sampling

Our research complied with all relevant regulations. Fieldwork and export of samples was approved by the local authorities, the Ministerio del Ambiente y los Recursos Naturales (MARENA), Nicaragua (permit numbers DGRNB-ACHL-0078. DRNB-IC-006-2007, No. 026-11007/DGAP, DGPN/DB-27-2010, DGPN-DB-IC-004-2013, DGPN/DB-011-2014, DGPN/DB-IC-015-2015). Fish were collected with gill nets or by harpooning in Nicaragua between 2003 and 2015. All specimens were photographed from the lateral view on site. The total number of individuals in this study is 120 (Source data): *A. sagittae* (*n* = 13), *A. xiloaensis* (*n* = 10), hybrids (*n* = 11), backcrosses (*n* = 21), and other Midas cichlid species in CL Xiloá, *A. viridis* (*n* = 19), *A. amarillo* (*n* = 21), and the outgroup *A. citrinellus* (*n* = 25) from Great Lake Managua.

### Re-sequencing data

Whole genome sequences^26^ are available in the European Nucleotide Archive (ENA) under accession PRJEB38173. Genomic sequences were obtained from libraries prepared using Illumina TruSeq DNA Nano kits (Illumina) aiming for 350 bp insert sizes. Genomic libraries were paired-end sequenced (2 × 150 bp) on a HiSeq 4000 or HiSeq X-Ten Illumina platform at the Beijing Genomics Institute (BGI, Shenzen), resulting in an average effective genome coverage of 25.6× ± 6.3× per individual. We used the Midas cichlid chromosome-level genome assembly (GenBank: JACBYM000000000.1) as reference genome^25^.

All detailed bioinformatics steps to map and filter sequences are described in ref. 26. Briefly, reads were mapped using *BWA mem v0.7.15*^46^ and variants and individual genotypes were called with *freebayes v1.1.0*^47^. Subsequently, hard filters were applied using the vcffilter script from the *vcflib* package v1.0.4 (https://github.com/vcflib/vcflib) to remove low-quality variant sites. Variant representation was normalized with *vt norm*^48^ and the python script in ref. 26 was applied to decompose multi-nucleotide variants into single nucleotide variants. Individual genotype calls based on a read depth smaller than five were set to missing for all downstream analyses. Genomic data handling and filtering steps were performed with *vcftools v.0.1.15*^49^ and *plink v.1.90/v. 2.00*^50^. All analyses were performed with data from all 24 chromosomes. To minimize the impact of potentially misassembled regions on downstream analyses the masking criteria used in ref. 26 were applied (removing sites with a sequencing coverage across all Midas cichlid samples more than four standard deviation above the mean, low mappability regions, regions within 5 bp of an indel and regions close to repetitive regions or gaps in the assembly). Finally, haplotypes were inferred by statistical phasing with *SHAPEIT2 v.2.r900*^51^.

### Exploratory genomic analyses

We estimated Weir & Cockerham F_st_ in non-overlapping 10-kb windows for pairwise comparisons between the parental species and the hybrids (–fst-window-size 10,000) using *vcftools* v0.1.15^49^, as well as quantified the deviation of observed from expected genome-wide heterozygosity (–het) on a per-individual basis using the inbreeding coefficient (F-statistic = 1 – observed heterozygosity/expected heterozygosity) only considering SNPs with a minimum coverage = 5 (–minDP). We subsampled a total of 175,942 SNPs for 95 Midas cichlid individuals in CL Xiloá using *vcftools*, with a minimum physical separation of 50 kb along the chromosomes and performed a Principal Component Analysis (PCA) using the R package *adegenet* v2.1.2^52^. Individual ancestry components were estimated using *Admixture* v1.3^53^ including 5-fold cross-validation (–cv=5). Finally, using a custom script (available in Figshare, link below), we extracted private high-frequency allele across the genome, when comparing all four Midas cichlid species and hybrids in CL Xiloá. We only considered SNPs with no missing data (–max-missing 1 in *vcftools*) at individual genotypes with minimum coverage of 5 (–minDP 5 in *vcftools*) and private alleles present in at least 50% of individuals per species and not detected in any other species from CL Xiloá. We fitted a linear model between private allele counts and sample size, using *lm* function in R. In addition, we also searched for these alleles in available whole genomes of Midas cichlids from other lakes (other 333 individuals, dataset taken from ref. 26).

### Phylogenetic inference and concordance factor calculations

A neighbor-joining tree (NJ) and a coalescent-based phylogenetic tree were inferred using 88,369 SNPs and including a total 240 alleles (i.e., 120 individuals) corresponding to the focal taxa *A. sagittae* + backcrosses (*n* = 27), *A. xiloaensis* + backcrosses (*n* = 17), hybrids (*n* = 11), *A. viridis* (*n* = 19), *A. amarillo* (*n* = 21), and *A. citrinellus* (from Great Lake Managua, *n* = 25) as outgroup. Phylogenetic inferences were performed using the NJ command and the coalescent-based program SVDquartets^54^ in PAUP* 4.0^55^. The analysis was run by sampling one million random quartets and calculating 200 bootstrap replicates to assess statistical support. To describe the level of SNP discordance along the whole genome, we applied the same matrix to the SNPsCF function^29^ to obtain concordance factors calculations for all possible 4-taxon combinations (15 species quartets in total). We sampled a total of 100 individual quartets (n.quartets = 100, between.species.only = TRUE) and using default values for all remaining settings.

### Hybridization tests

First, to explore the level of genetic recombination in hybrids, we extracted a total of 52 biallelic SNPs (minimum depth = 5, no missing data) that are fully alternatively fixed between the parental species *A. sagittae* (13 individuals) and *A. xiloaensis* (10 individuals) and coded them as 0 = homozygous parental species A, 1 = heterozygous, and 2 = homozygous for the alternative variant in parental species B. No minimum physical distance between fixed SNPs was applied, since in this exploratory analysis we intended to analyze potential recombination of closely located variants. These variants were used to plot the matrix shown in Fig. 2a and to calculate hybrid indices using the introgress v1.2.3 R package^56^. In addition, we also extracted a total of 615 SNPs along the genome, using a relaxed allele frequency in one parental species ≥95% while ≤5% in the other (Supplementary Fig. 10). From this matrix, we subsampled only SNPs with a minimum separation of 50 kb and obtained a total of 148 SNPs, which we analyzed with the R package HIest v2.0^32^ using as genotype matrix (G) and their respective allele frequencies (P). In addition, ancestry tract length distributions along the whole genomes were reconstructed using ChromoPainter v2^57^. Using the phased genotypes, we generated input files for each chromosome including the 10 individuals with the highest genome-wide sequencing coverage from each of the two parental species as well as the 11 hybrid samples and 21 backcrosses. We omitted all variable sites that were masked in the reference genome or had more than 20% genotypes represented as either missing or with less than 5× sequencing coverage. To account for recombination rate variation, we included a genetic map based on the window-wise estimates of recombination rates (see Recombination rate estimation in ref. 26). We specified equal prior probabilities for each donor population and ran ChromoPainter by estimating the average switch rate parameter and global mutation probability using 10 E-M iterations to obtain copy probabilities for each hybrid and backcross haplotype. We calculated summary statistics of ancestry tract length distributions for hybrids and backcrosses and fitted a linear model to statistically test differences in ancestry tract length in relation to groups and donors and their interactions (ancestry tract length ~ groups * donor).

We statistically tested hybridization and estimated the inheritance parameter (γ) using the HyDe program^58^. HyDe uses phylogenetic invariants, similar to the D-statistic^59^, to assess statistically significant evidence for hybridization. Specifically, we employed 88,369 SNPs considering 55 individuals in the python script *run_hyde.py* to test the triplet comprising the two parental species and the hybrids.

### Demographic models

Furthermore, we applied the multiple sequentially Markovian coalescent model implemented in MSMC v2.1.2^33^ to estimate effective population sizes and gene flow through time. We used the three individuals with the highest sequencing coverage per population (i.e., *A. xiloaensis*, *A. sagittae*, and hybrids) to calculate the relative cross-coalescence rate (RCCR) as a proxy for gene flow between population pairs, only including sites with a minimum depth of 5 and genotyped in 80% of individuals for each analyzed population. For the MSMC2 runs, we applied the default settings, including 10 bootstrap replicates, for the number of iterations and the time segment pattern and scaled the output into years/number of individuals by assuming a mutation rate of 3.5 × 10^−9 ^^36^ per site per generation and generation time of 1.5 years. In addition, demographic parameters were estimated using the coalescent-with-introgression model implemented in MSci program^34^. This program requires long-sequence loci (instead of SNPs), thus we reconstructed 1025 phased loci of 2 kb length each for 34 ingroup individuals (68 haplotypes). Loci were selected randomly from the reference genome, requiring a minimum distance of 20 kb between loci, a minimum distance of 5 kb to any annotated exon, and 2000 unmasked sites within a physical distance of <3 kb. In the Msci control file, we specified an inverse gamma prior using a diffuse alpha, and the beta parameter was set based on the same mutation rate as used for MSMC2 (see above)^36^. Accordingly, hyperprior parameters were specified as *θ* (*α* = 3, *β* = 2.31 × 10^−6^) and *τ* (*α* = 3, *β* = 5.264 × 10^−6^). The species tree was fixed (speciestree = 0) with finetune settings = 1. The Markov Chain Monte Carlo (MCMC) algorithm was run for 500,000 steps, sampling every 50 steps with a 10% burn-in period.

### Gene ontology

Using ShinyGo v0.76^60^, gene ontology (GO) term enrichment analyses were performed for the annotations of private alleles in hybrids (14 different genes), as well as fixed SNPs in parental species (18 different genes). We used gene abbreviations and ensemble gene IDs, the Nile Tilapia reference genome, and an FDR cutoff = 0.2 to search for GO Biological Processes. All remaining settings were kept at default. We show results for the 20 most significantly enriched terms.

### Morphometric analyses

For geometric morphometric analyses a total of 95 photographs were included, corresponding to all individuals used for genomic analyses in CL Xiloá. The configurations of points used in morphometric analyses of body shape (Supplementary Fig. 15) comprised 14 fixed landmarks, seven semi-landmarks, and six helper points. Helper points are used to help the alignment of the other landmarks but are later excluded from the analyses as they do not provide additional information. Points were digitized on photographs using *tpsDig v.2.32*^61^. All further analyses were performed with the *geomorph v3.0.6*R package^62^. Landmarks were aligned using a full Procrustes superimposition with the function *gpagen*. Linear distances were obtained using the function *interlandmark* (Supplementary Fig. 16), including standard length (LM1-9), body depth (LM3-15), caudal peduncle depth (LM6-12, LM7-11, and LM8-10), pectoral fin width (LM18-19), and eye width (LM20-21). In addition, head angle was obtained from the angle formed in the intersection of lines connecting LM1-15 and LM1-3. Several exploratory analyses were performed. In all cases, allometry was controlled for by regressing shape variables on body standard length (taken from LM1 to LM9; Supplementary Fig. 16) and using regression residuals in subsequent analyses. Principal component analyses (PCA) were performed using the *prcomp* R function. Procrustes distances between groups of interest were performed using the *procD.lm* function. Consensus shapes were obtained using the *mshape* function, and plots of deviation were constructed with the *plotRefToTarget* function. Discriminant function for comparison of different groups of interest was performed using the *lda* function of the *MASS v7.3* library^63^. All linear correlations (Fig. 3b, c; Supplementary Figs. 18 and 19) were performed using the *lm* R function.

### Trophic analyses

Stable isotope ratios (SIA) of carbon (δ^13^C) and nitrogen (δ^15^N) were calculated from white muscle tissue (taken from severed heads, dorso-posterior near the lateral line) in the Limnological Institute at the University of Konstanz. Individuals were analyzed by gas chromatography combustion isotope ratio mass spectrometry (GC-C-IRMS). All individuals were adults that had been collected in different expeditions of the Meyer lab to Nicaragua (from 2010 to 2014) with 28 individuals taken from ref. 27, plus one individual from Rometsch et al. (*in prep*.) and 30 new individuals. In total, we collected SIA information from 9 hybrids, 10 *A. sagittae*, 11 *A. xiloaensis*, 19 *A. viridis*, and 10 *A. amarillo*. For all individuals, species identification was first based on their morphology and later confirmed based on genomic data, either included in this study (whole genome sequences) or from a previous study using RADseq data^25^. A MANCOVA test in R was performed to test for species differences. Due to a significant contribution of variation associated with sampling location and year among field trips, we used location and year of collection as co-variables in linear models (*lm* function) and MANCOVA (*manova* function) tests to correct for potential differences associated with changing environmental conditions^22^.

### Reporting summary

Further information on research design is available in the Nature Research Reporting Summary linked to this article.

## Supplementary information

Supplementary Information

Reporting Summary

## Data Availability

The 120 raw whole genome sequences used in this study in the form of unmapped BAM files are available in the European Nucleotide Archive (ENA) under accession PRJEB38173^26^. Source data are provided with this paper for the processed whole genome sequences and the raw geometric morphometric dataset (Source data file 2 available on FigShare, https://figshare.com/projects/Early_stages_of_sympatric_homoploid_hybrid_speciation_in_crater_lake_cichlid_fishes/132428). The stable isotope data are provided in Source data file 1. Source data are provided with this paper.

## Source data

Source Data

## Code availability

The custom computer code used in this study is provided in Source Data file 2, available on FigShare (https://figshare.com/projects/Early_stages_of_sympatric_homoploid_hybrid_speciation_in_crater_lake_cichlid_fishes/132428).
