# Supplementary material for: Early stages of sympatric homoploid hybrid speciation in crater lake cichlid fishes
Source: Nat Commun. 2022 Oct 6;13:5893. doi: 10.1038/s41467-022-33319-4 (PMC9537415; doi:10.1038/s41467-022-33319-4)
Supplement: Supplementary file 1 — Supplementary Information [file 41467_2022_33319_MOESM1_ESM.pdf]

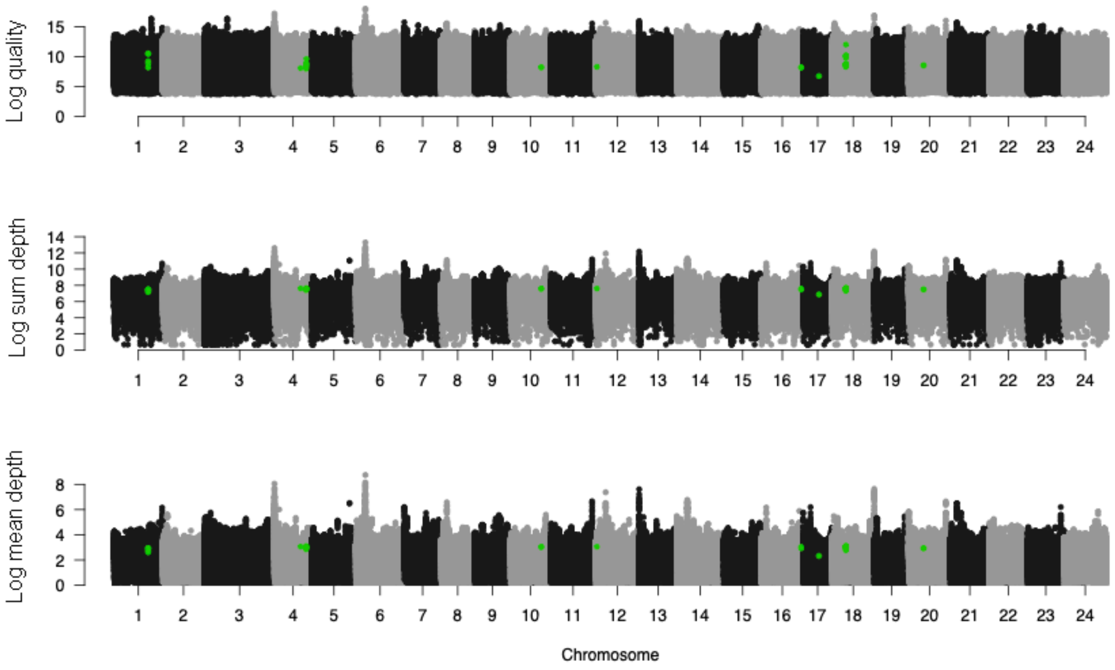

Supplementary Fig. 1: Manhattan plots showing information obtained with vcftools (using all Midas in CL Xiloá, n=95, shown in log scale) including per site SNP quality (--site-quality, QUAL), depth per site summed across all individuals (--site-depth, SUM_DEPTH) and the mean in depth per site averaged across all individuals (--site-mean-depth, MEAN_DEPTH). Green dots are sites found as private SNPs in hybrids (n=11, frequency cut off = 0.5, see also Supplementary Table 3). Source Data is provided in Source Data file 2.

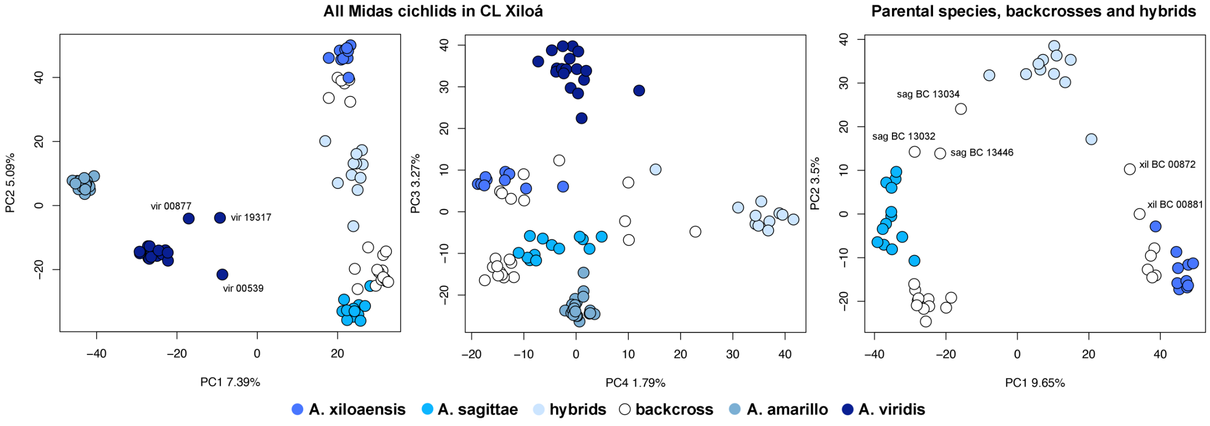

Supplementary Fig. 2: First two plots on the left are 2D scatter plots using genetic PC1-2 and PC3-4, respectively, based on 175,942 SNPs for all four Midas cichlid species and hybrids in CL Xiloá (3D plots shown in Fig. 1 a-b in the main manuscript). 2D PCA based on only parental species *A. sagittae* and *A. xiloaensis*, hybrids, and backcrosses is presented on the right. IDs of three *A. viridis* that deviate from the species cloud and five backcrosses closer to hybrids are shown. Source data are provided as Source Data file 1.

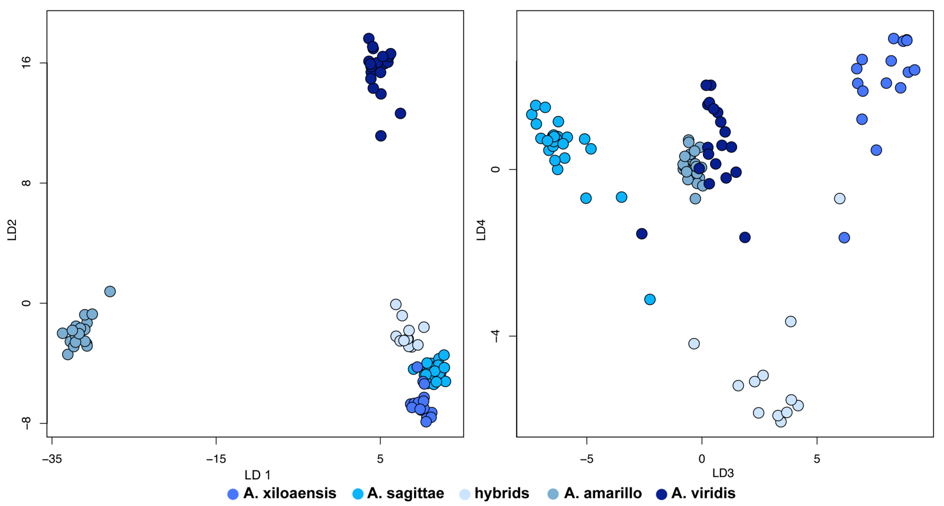

Supplementary Fig. 3: Scatter plot of LD1 - LD2 (left) and L3 – LD4 (right) scores obtained from a linear discriminant function based on genomic data (175,942 SNPs) including the two parental species and their backcrosses, hybrids and the remaining two Midas cichlid species in CL Xiloá. Source data are provided as Source Data file 1.

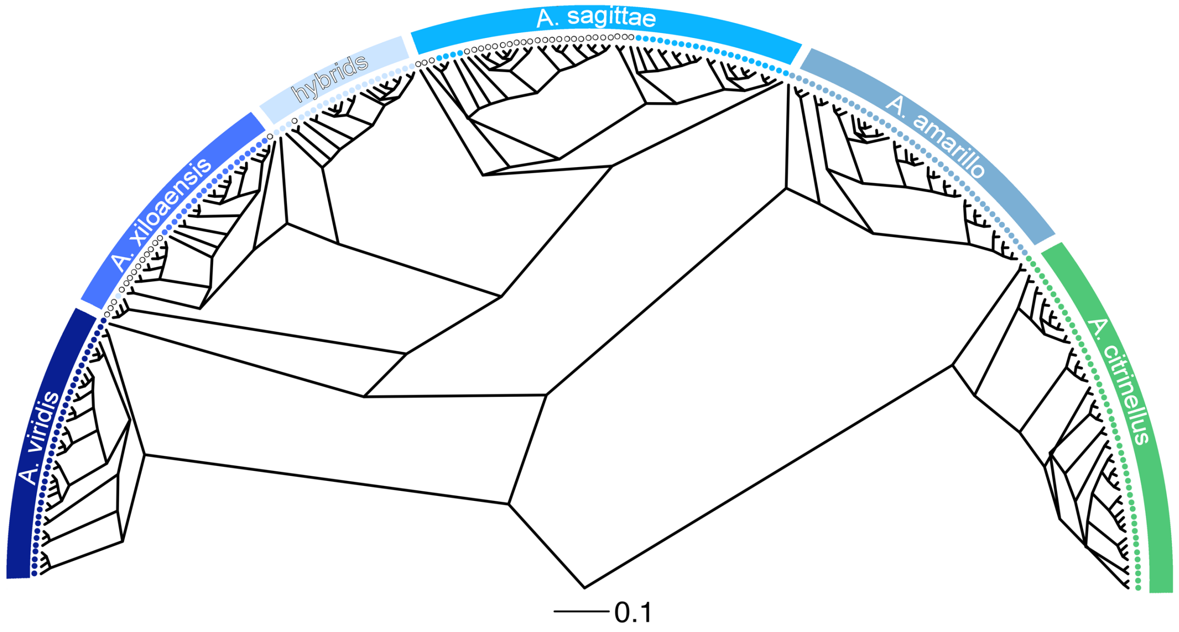

Supplementary Fig. 4: Neighbor joining tree based on 88,369 SNPs and 240 alleles (extracted from 120 diploid individuals). Colored circles on the tips represent individuals per species as classified in clade legends, as well as backrcosses in white. One haplotype of the xiloeansis backcross 13034 is nested within the hybrid clade, and one haplotype of hybrid 00882 is nested within pure A. xiloaensis. Source Data is provided in Source Data file 2.

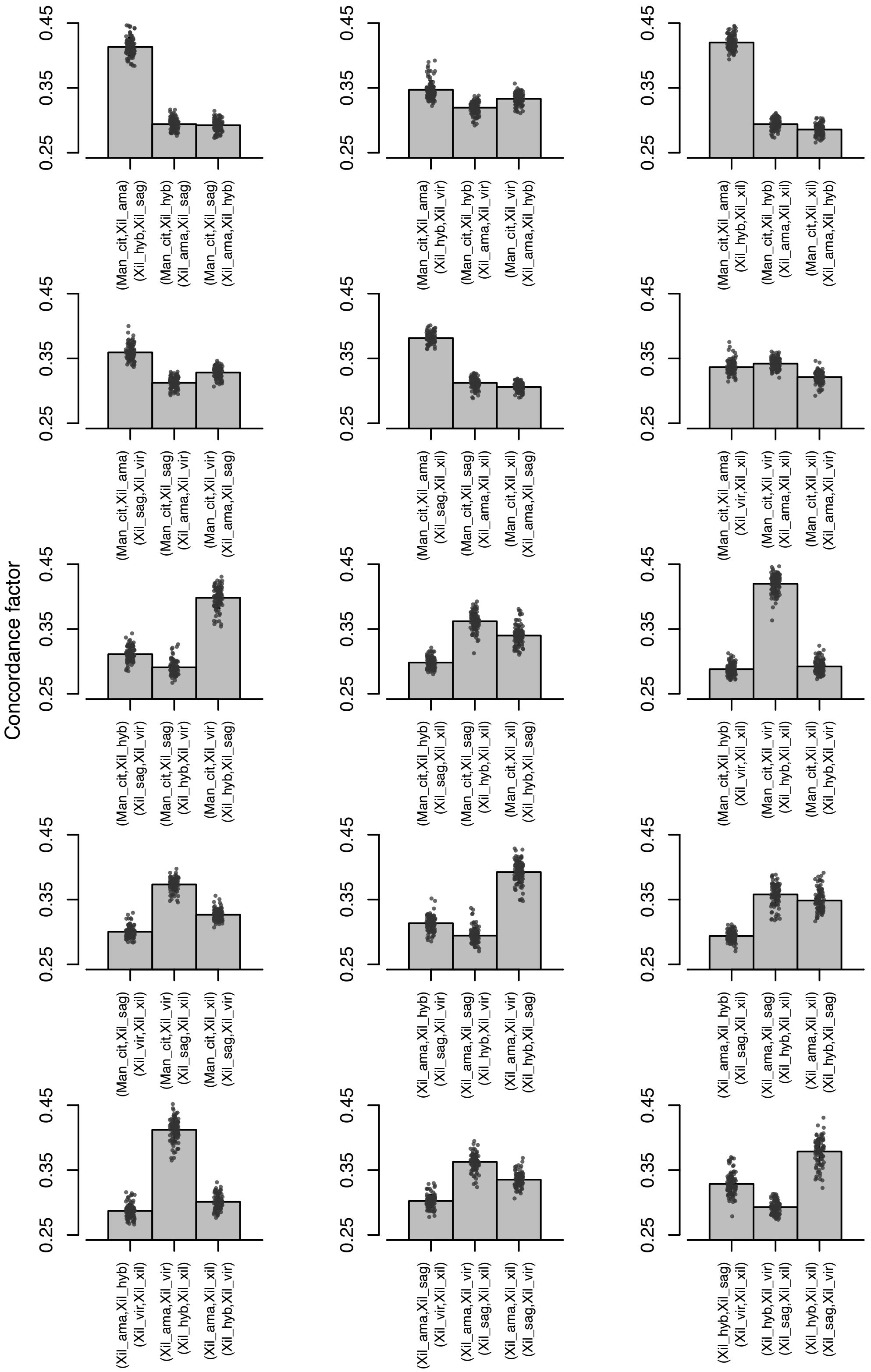

Supplementary Fig. 5: Mean concordance factors (CF = proportion of genome wide SNPs recovering each of the three possible alternative topologies for a given species quartet) based on 100 individual quartets with 88,369 SNPs for all 15 possible quartets in Midas cichids in CL Xiloá and one outgroup. Individual data points are shown on the bars. Abbreviations: Xil_xil = A. xiloaensis; Xil_sag = A. sagittae; Xil_hyb = hybrids; Xil_vir = A. viridis; Xil_ama = A. amarillo; Man_cit = A. citrinellus (outgroup). Source data are provided as Source Data file 1.

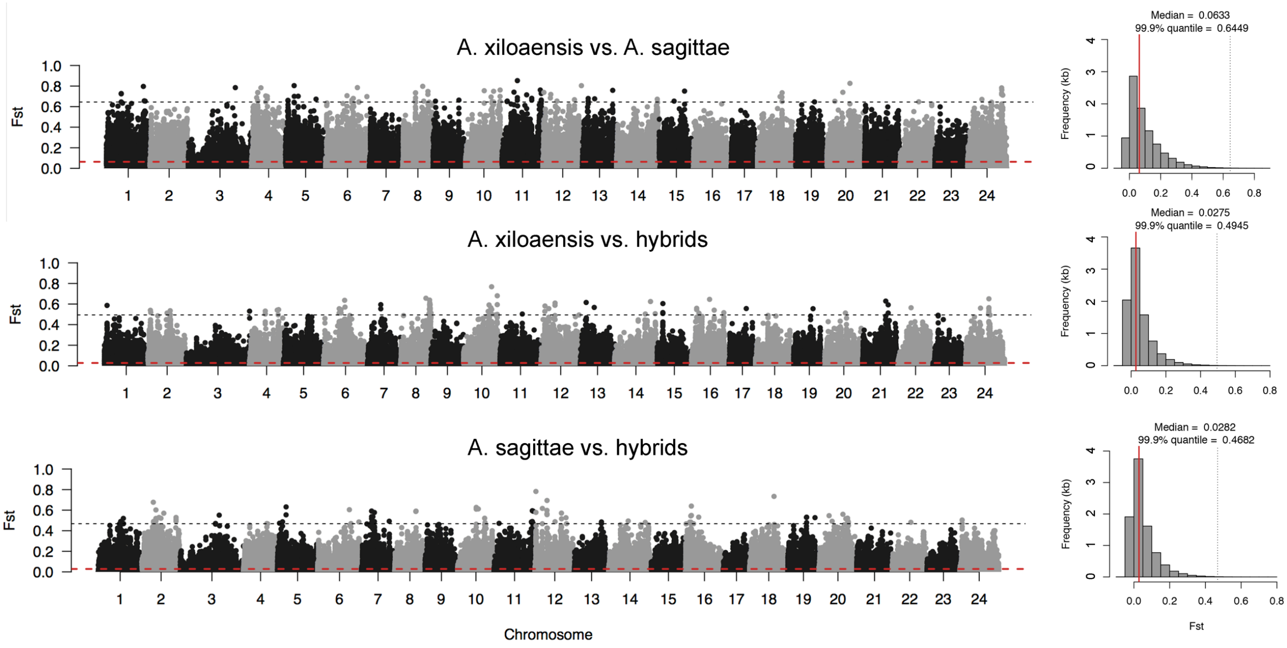

Supplementary Fig. 6: F_{st} calculation along the genome based on 10-kb windows. The median and 99.9% quantiles are shown by the red and black dotted lines, respectively. Source data are provided in vcf format in Source Data file 2.

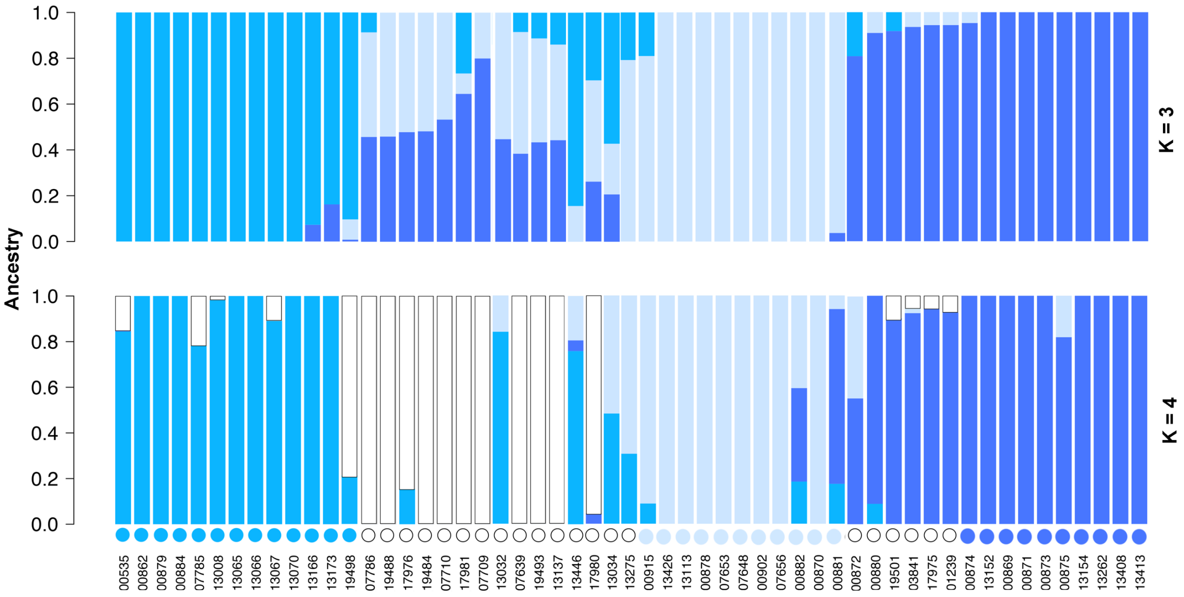

Supplementary Fig. 7: Admixture plots constructed using 175,942 SNPs and assuming K=3 (CV=0.1558) and K=4 (CV=0.16514). Source data are provided as Source Data file 1.

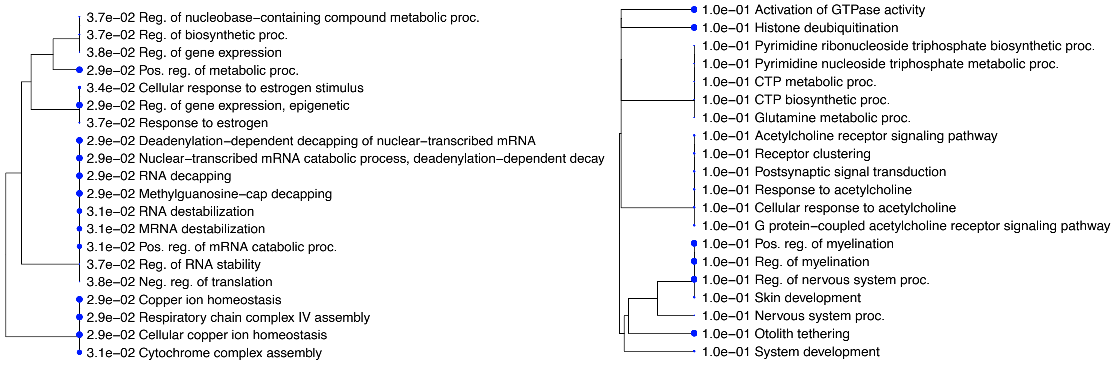

Supplementary Fig. 8: Hierarchical clustering tree of gene ontology (GO) terms fore gene annotations in concordance with Supplementary Table 4 and 7. Pathways with many shared genes are clustered together. Bigger dots indicate more significant p-values in false discovery rate (FDR). Left, results based on private alleles in hybrids, involving different GO functions for biological processes, mainly clustering in four groups: (i) respiratory functions, (ii) different pathways for RNA degradation, (iii) response to estrogen stimulus and gene expression and (iv) related to metabolic processes. Right, results for GO enrichment analysis for fixed SNPs in parental species, including several different biological processes associated to them, including pyrimidine and CTP metabolism, as well nervous system processes.

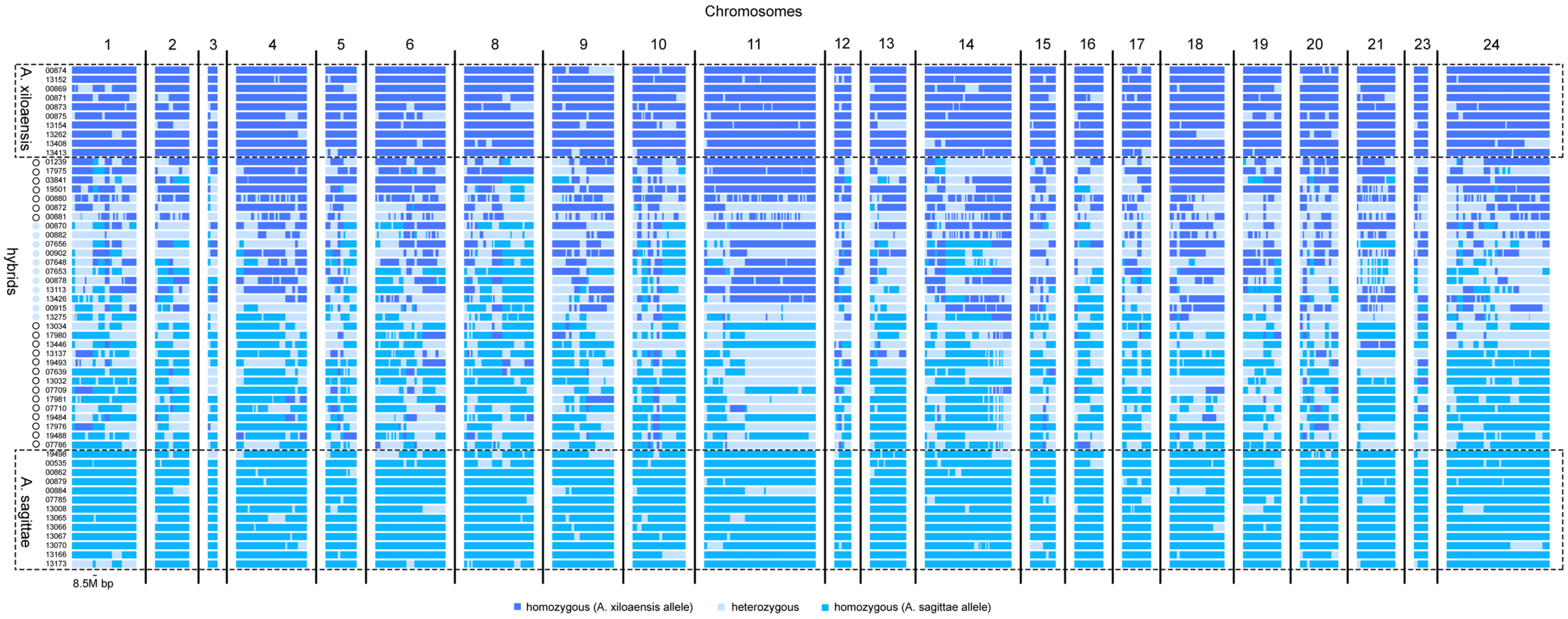

Supplementary Fig. 9: Matrix comparing hybrid genotypes at 615 SNPs under 95% fixation rate frequency between the parental species. Separation of SNP columns is in log-scale according to their physical distance within each chromosome (scale shown in chromosome 1, bottom). Source data is available in Source Data file 1.

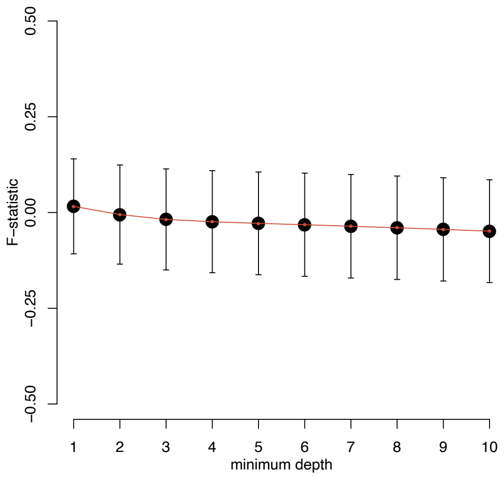

Supplemantary Fig. 10: Mean and standard deviation (n = 55 biologically independent individuals) of inbreeding coffecient (F-statistic = 1 – observed heterozygosity / expected heterozygosity) calculated based on different minimum depth filters applied to the genomic data (--minDP 1 to 10 in vcftools, no missing data filter applied in order to explore the impact of low coverage affecting calculated heterozygosyty). No significant difference are found in any comparisons between DP = 2 to 10 (two sided F-test; F = 0.6795 on 8 and 486 DF, p-value 0.7098). Source data are provided as Source Data file 1.

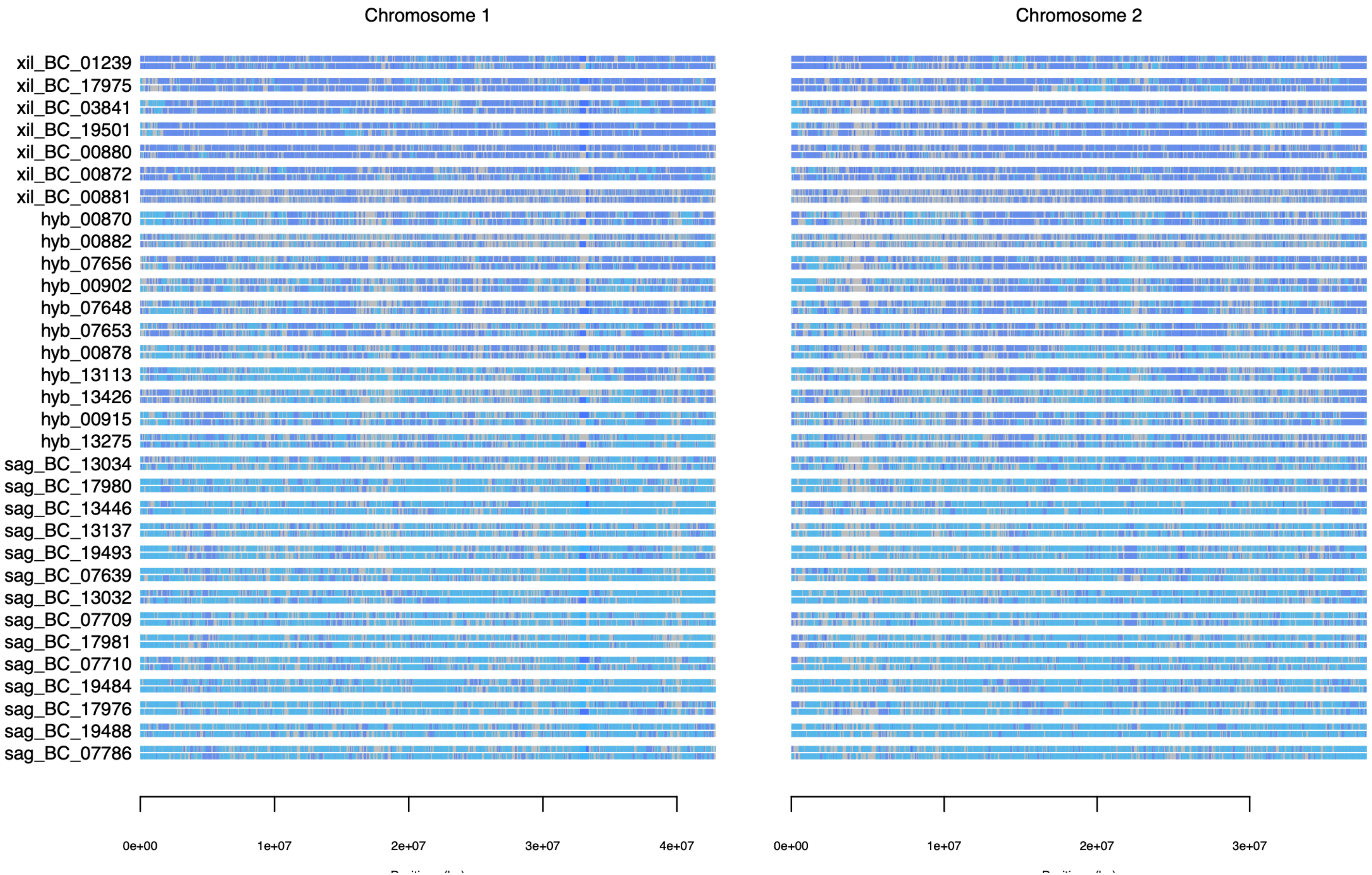

Supplementary Fig. 11: Estimate of ancestry with chromopainter for hybrid individuals + backrosses across their 24 chromosomes inferred as *A. sagittae* (light blue) or *A. xiloaensis* (dark blue) with probablity >0.8. Gray represents uncertain ancestry (probability <0.8). Source data are provided in Source Data file 2.This figure continues below.

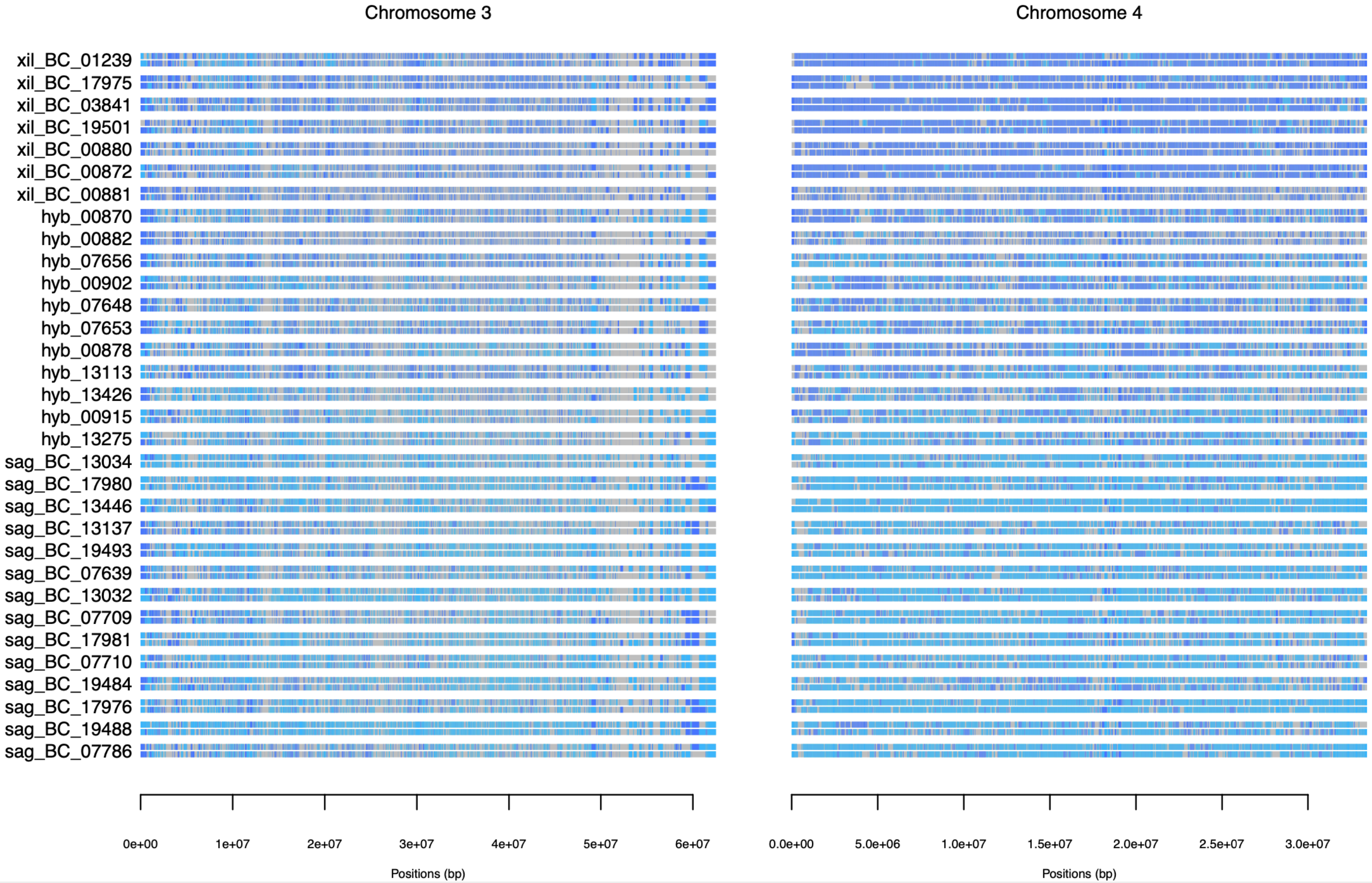

Supplementary Fig. 11 (continuation): Estimate of ancestry with chromopainter for 11 hybrid individuals across their 24 chromosomes inferred as *A. sagittae* (light blue) or *A. xiloaensis* (dark blue) with probability >0.8. Gray represents uncertain ancestry (probability <0.8). Source data are provided in Source Data file 2. This figure continues below.

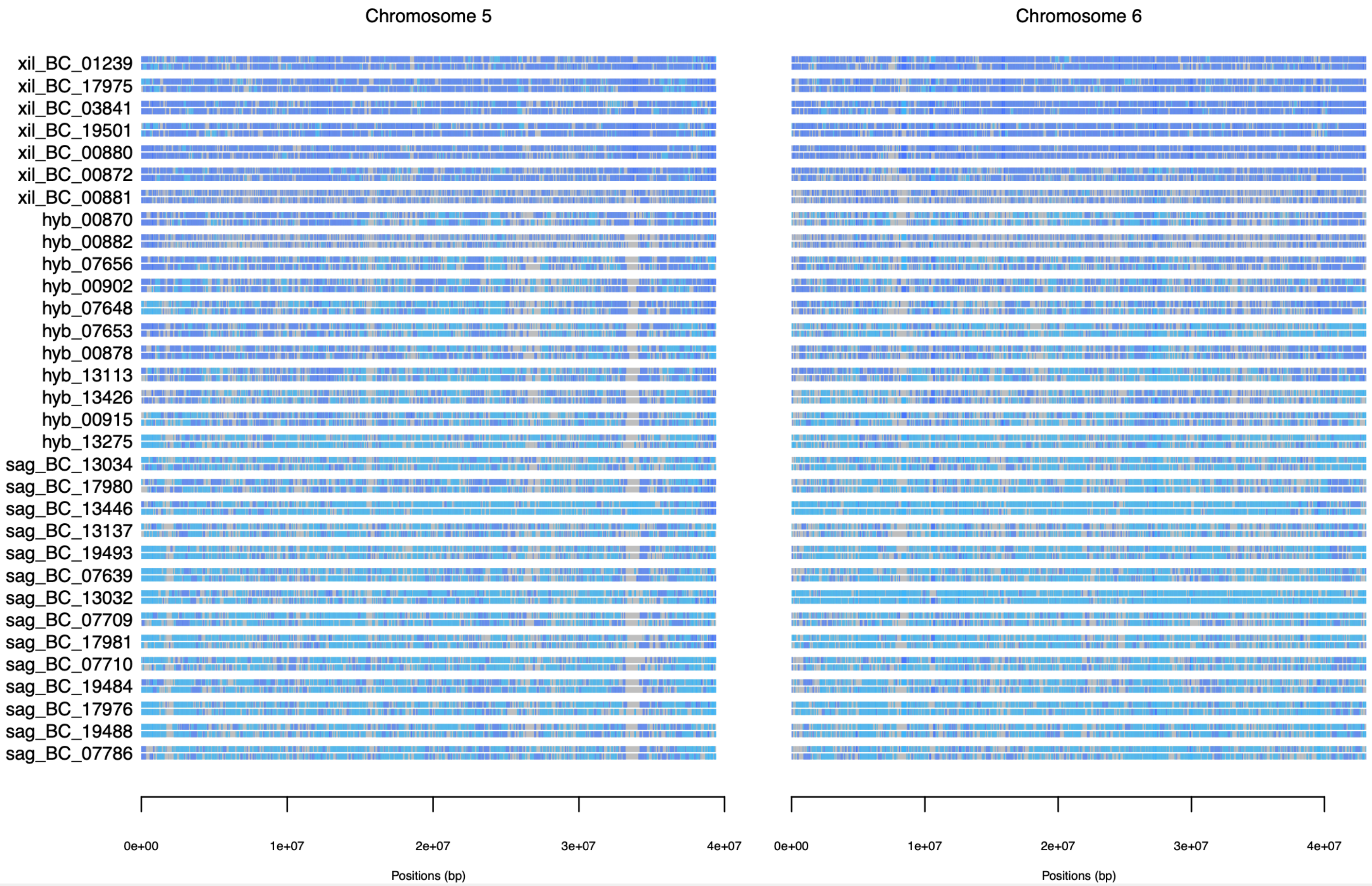

Supplementary Fig. 11 (continuation): Estimate of ancestry with chromopainter for hybrid individuals + backrosses across their 24 chromosomes inferred as *A. sagittae* (light blue) or *A. xiloaensis* (dark blue) with probability >0.8. Gray represents uncertain ancestry (probability <0.8). Source data are provided in Source Data file 2. This figure continues below.

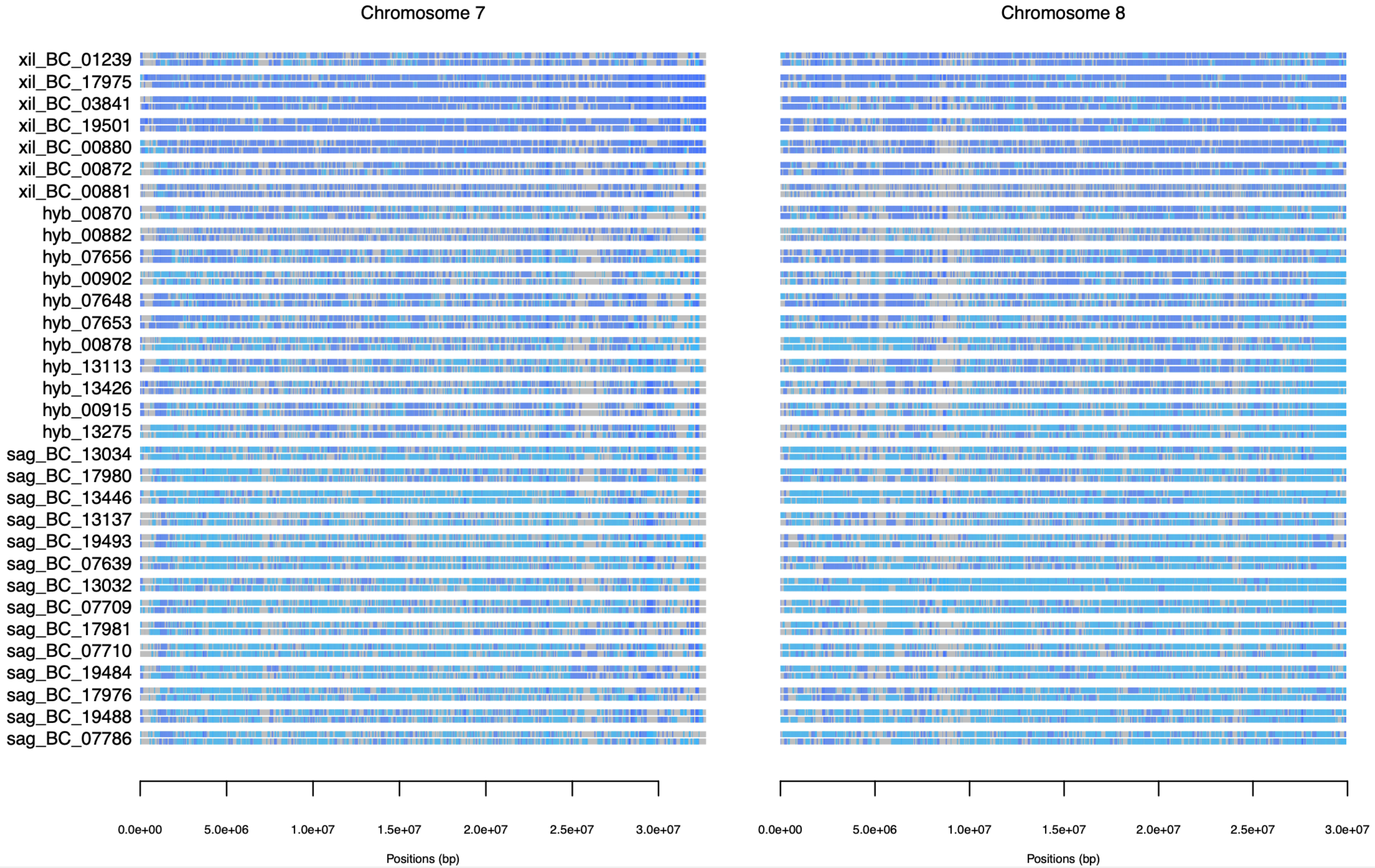

Supplementary Fig. 11 (continuation): Estimate of ancestry with chromopainter for hybrid individuals + backrosses across their 24 chromosomes inferred as *A. sagittae* (light blue) or *A. xiloaensis* (dark blue) with probability >0.8. Gray represents uncertain ancestry (probability <0.8). Source data are provided in Source Data file 2. This figure continues below.

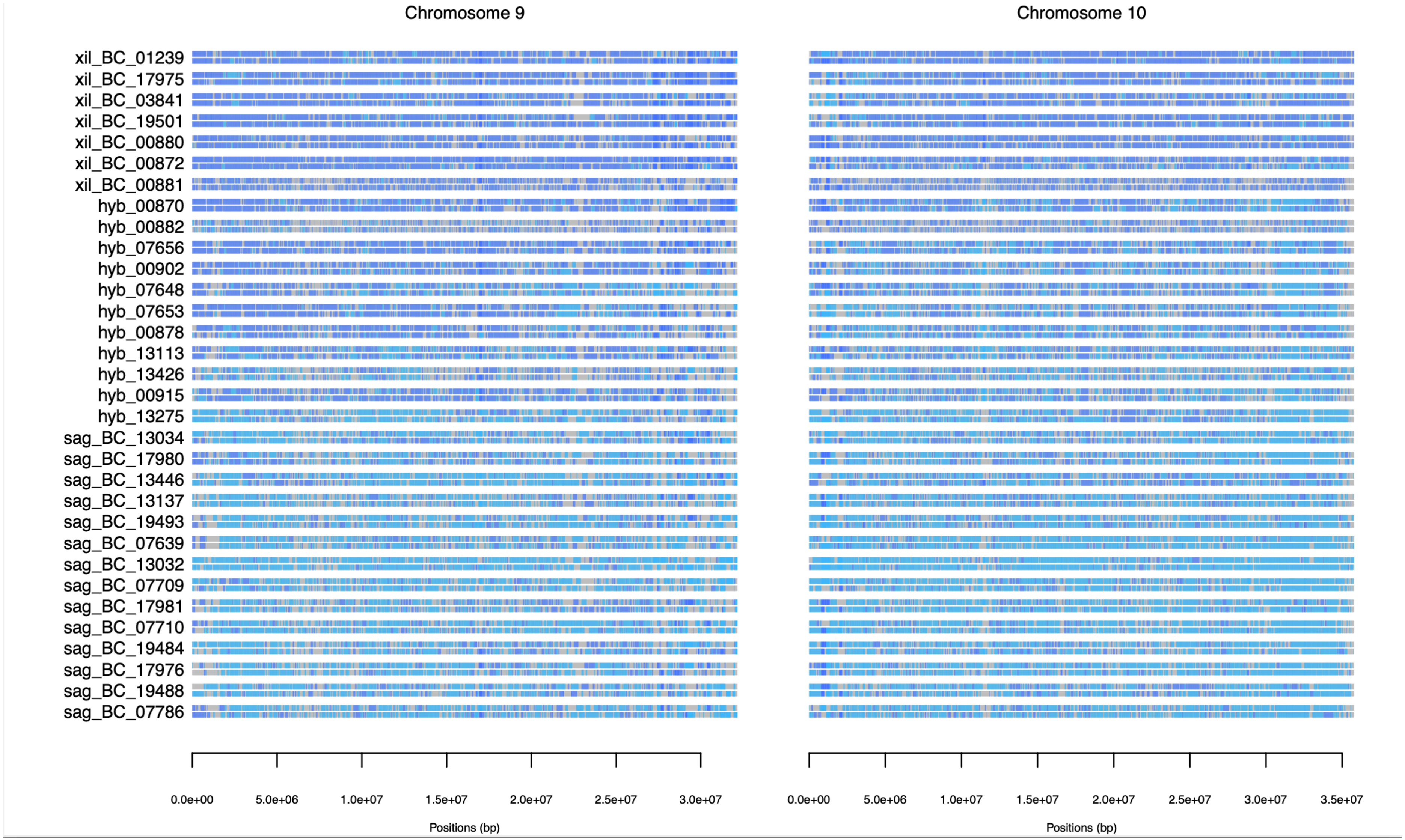

Supplementary Fig. 11 (continuation): Estimate of ancestry with chromopainter for hybrid individuals + backrosses across their 24 chromosomes inferred as *A. sagittae* (light blue) or *A. xiloaensis* (dark blue) with probability >0.8. Gray represents uncertain ancestry (probability <0.8). Source data are provided in Source Data file 2. This figure continues below.

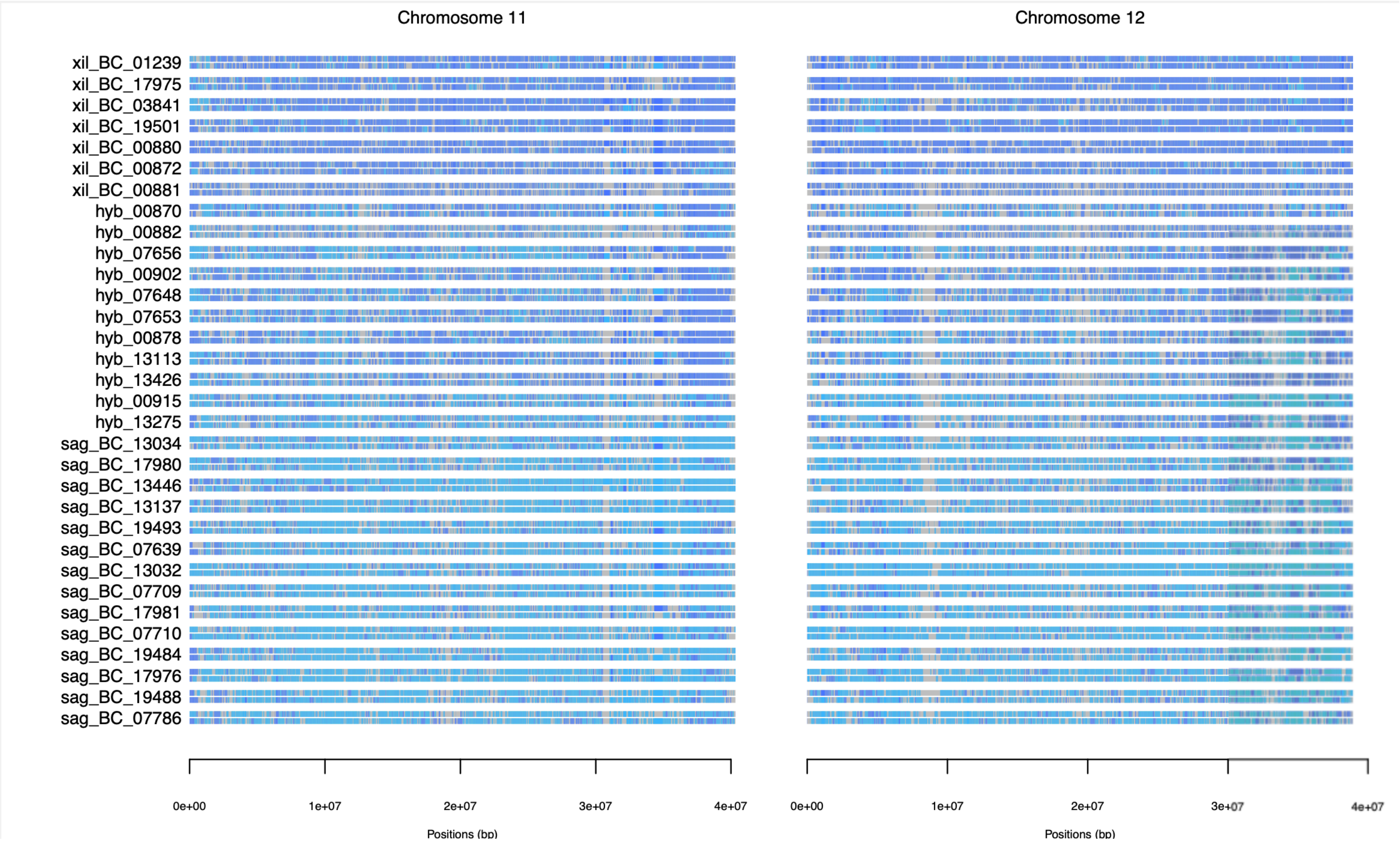

Supplementary Fig. 11 (continuation): Estimate of ancestry with chromopainter for hybrid individuals + backrosses across their 24 chromosomes inferred as *A. sagittae* (light blue) or *A. xiloaensis* (dark blue) with probability >0.8. Gray represent uncertain ancestry (probability <0.8). Source data are provided in Source Data file 2. This figure continues below.

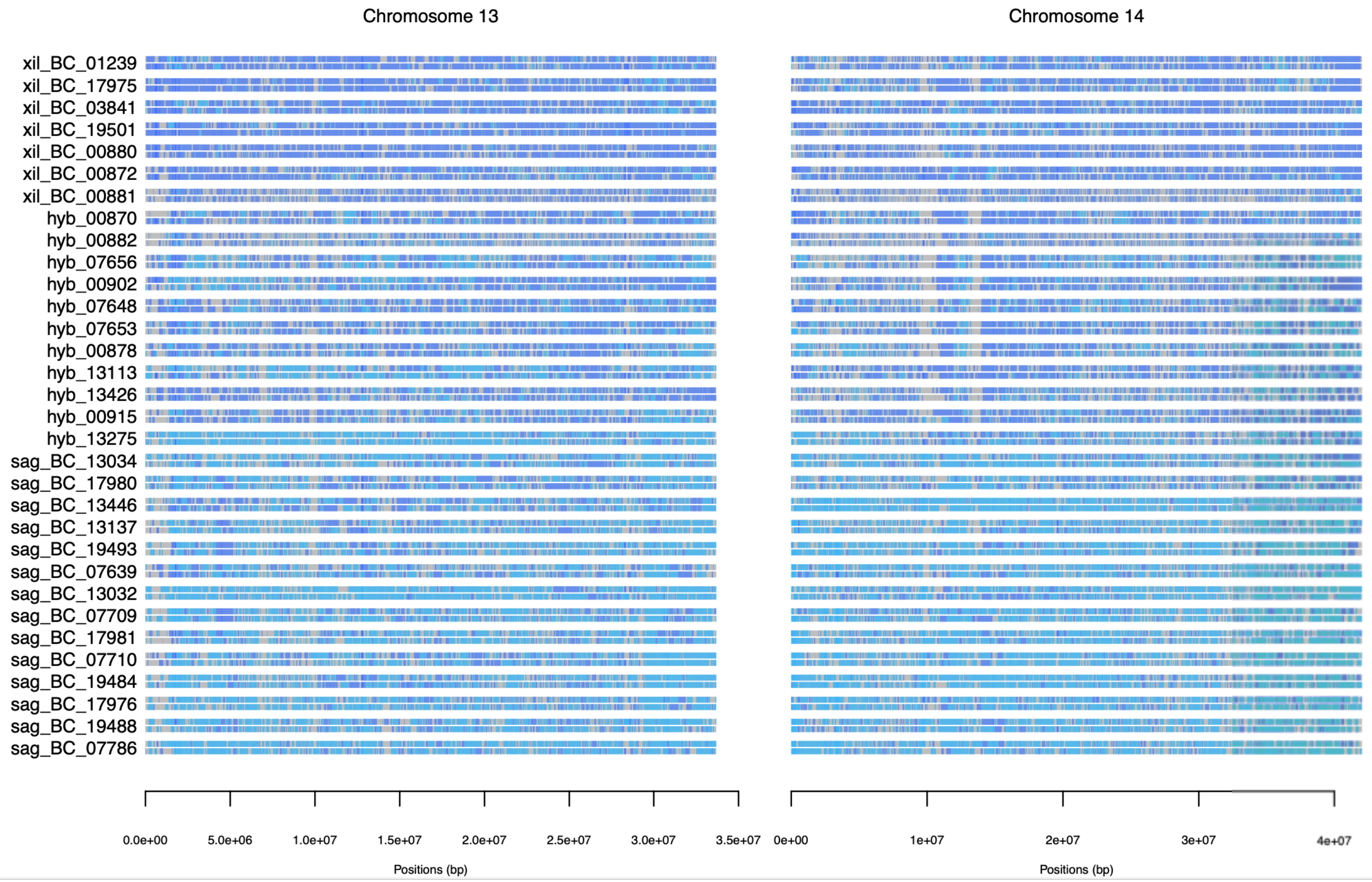

Supplementary Fig. 11 (continuation): Estimate of ancestry with chromopainter for hybrid individuals + backrosses across their 24 chromosomes inferred as *A. sagittae* (light blue) or *A. xiloaensis* (dark blue) with probability >0.8. Gray represents uncertain ancestry (probability <0.8). Source data are provided in Source Data file 2. This figure continues below.

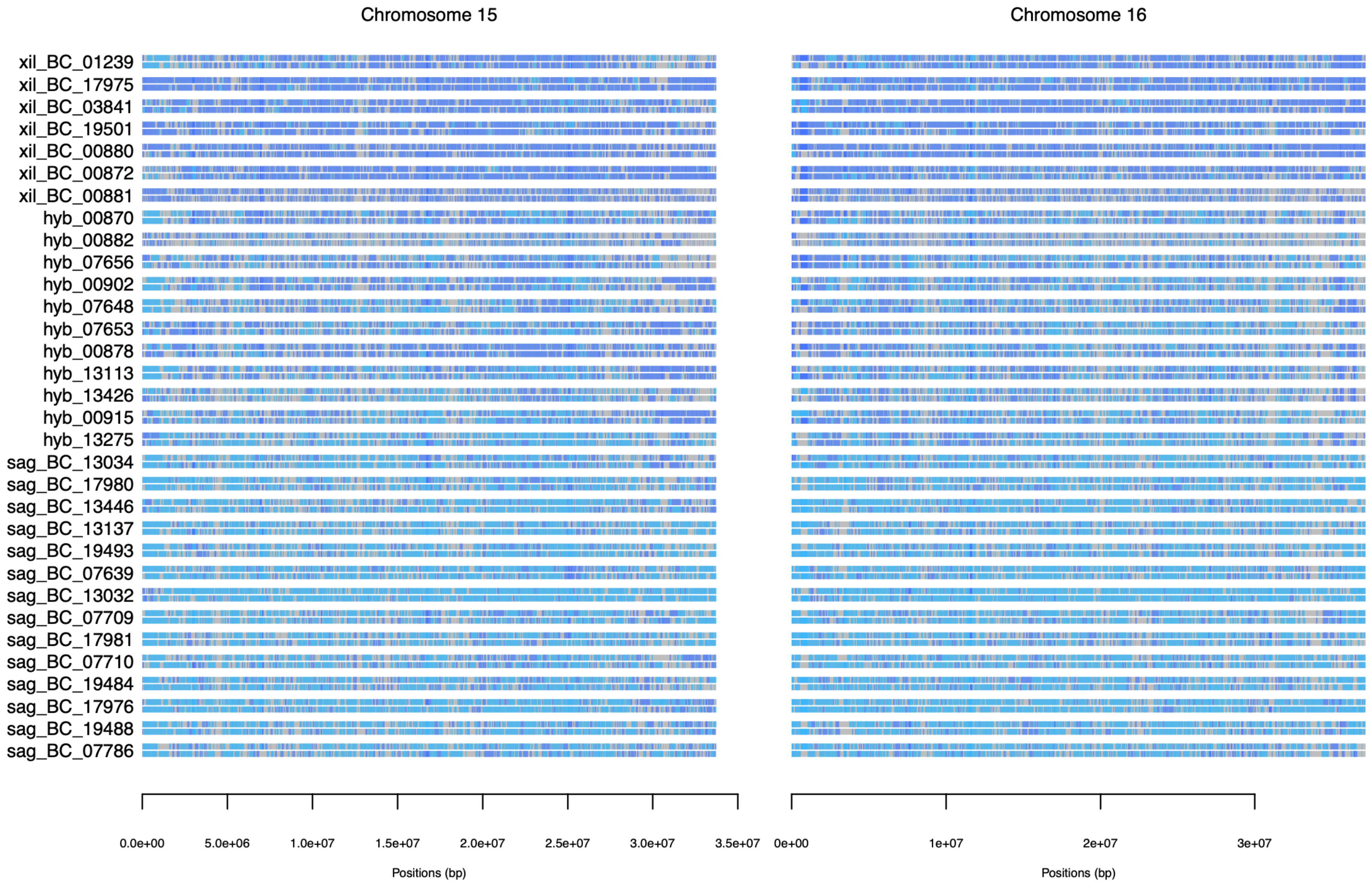

Supplementary Fig. 11 (continuation): Estimate of ancestry with chromopainter for hybrid individuals + backrosses across their 24 chromosomes inferred as *A. sagittae* (light blue) or *A. xiloaensis* (dark blue) with probability >0.8. Gray represents uncertain ancestry (probability <0.8). Source data are provided in Source Data file 2. This figure continues below.

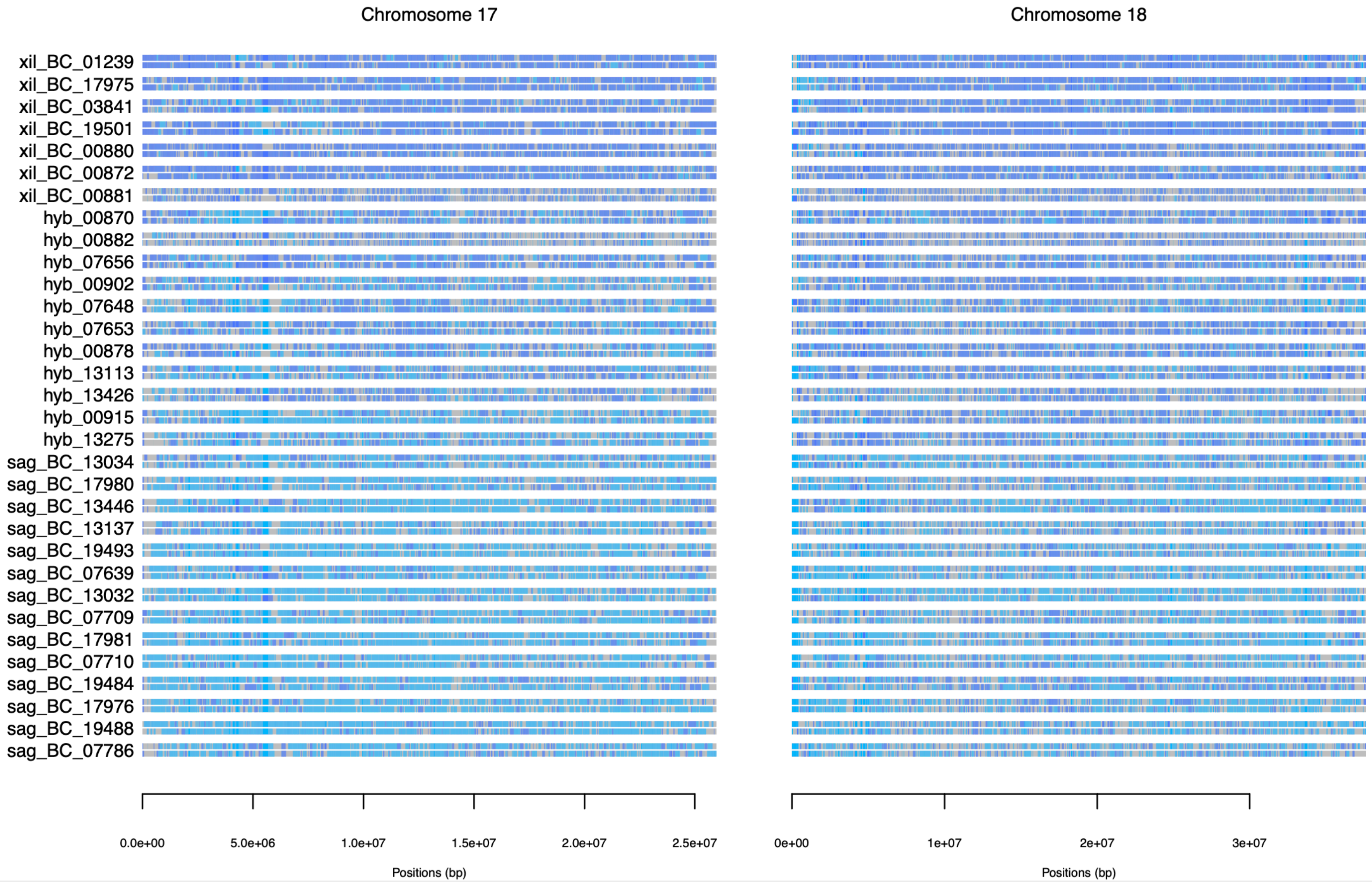

Supplementary Fig. 11 (continuation): Estimate of ancestry with chromopainter for hybrid individuals + backrosses across their 24 chromosomes inferred as *A. sagittae* (light blue) or *A. xiloaensis* (dark blue) with probability >0.8. Gray represents uncertain ancestry (probability <0.8). Source data are provided in Source Data file 2. This figure continues below.

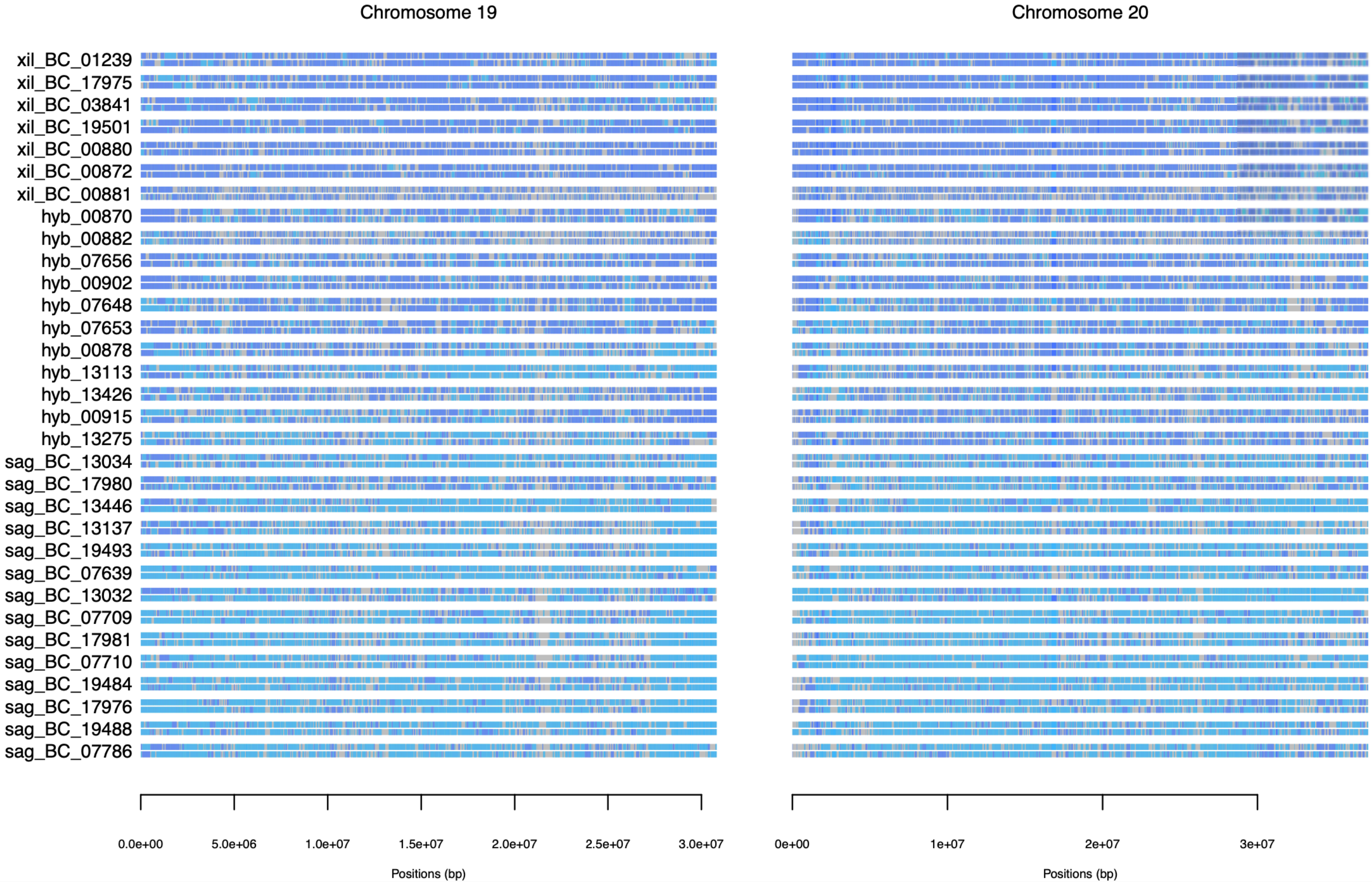

Supplementary Fig. 11 (continuation): Estimate of ancestry with chromopainter for hybrid individuals + backrosses across their 24 chromosomes inferred as *A. sagittae* (light blue) or *A. xiloaensis* (dark blue) with probability >0.8. Gray represents uncertain ancestry (probability <0.8). Source data are provided in Source Data file 2. This figure continues below.

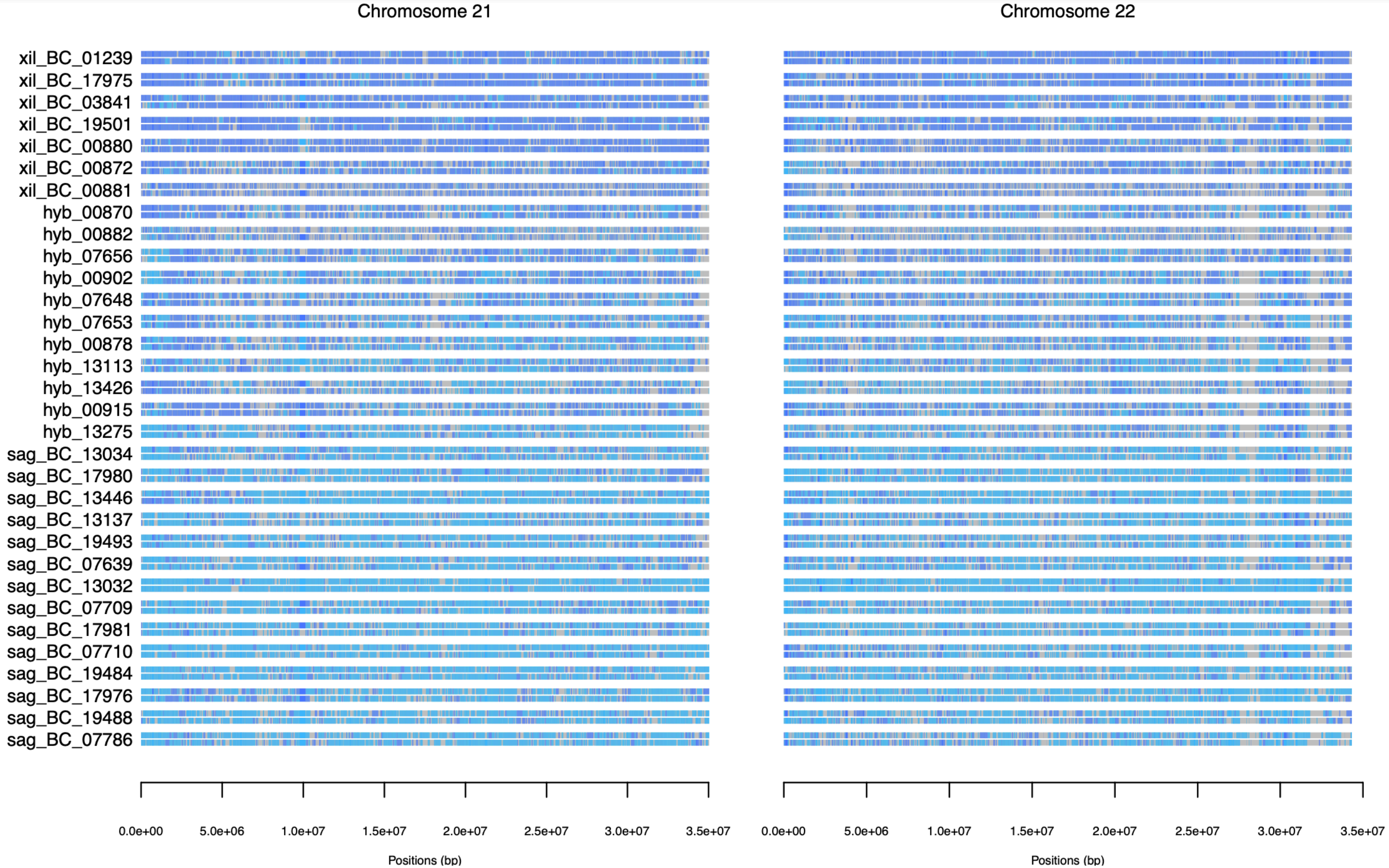

Supplementary Fig. 11 (continuation): Estimate of ancestry with chromopainter for hybrid individuals + backrosses across their 24 chromosomes inferred as *A. sagittae* (light blue) or *A. xiloaensis* (dark blue) with probability >0.8. Gray represents uncertain ancestry (probability <0.8). Source data are provided in Source Data file 2. This figure continues below.

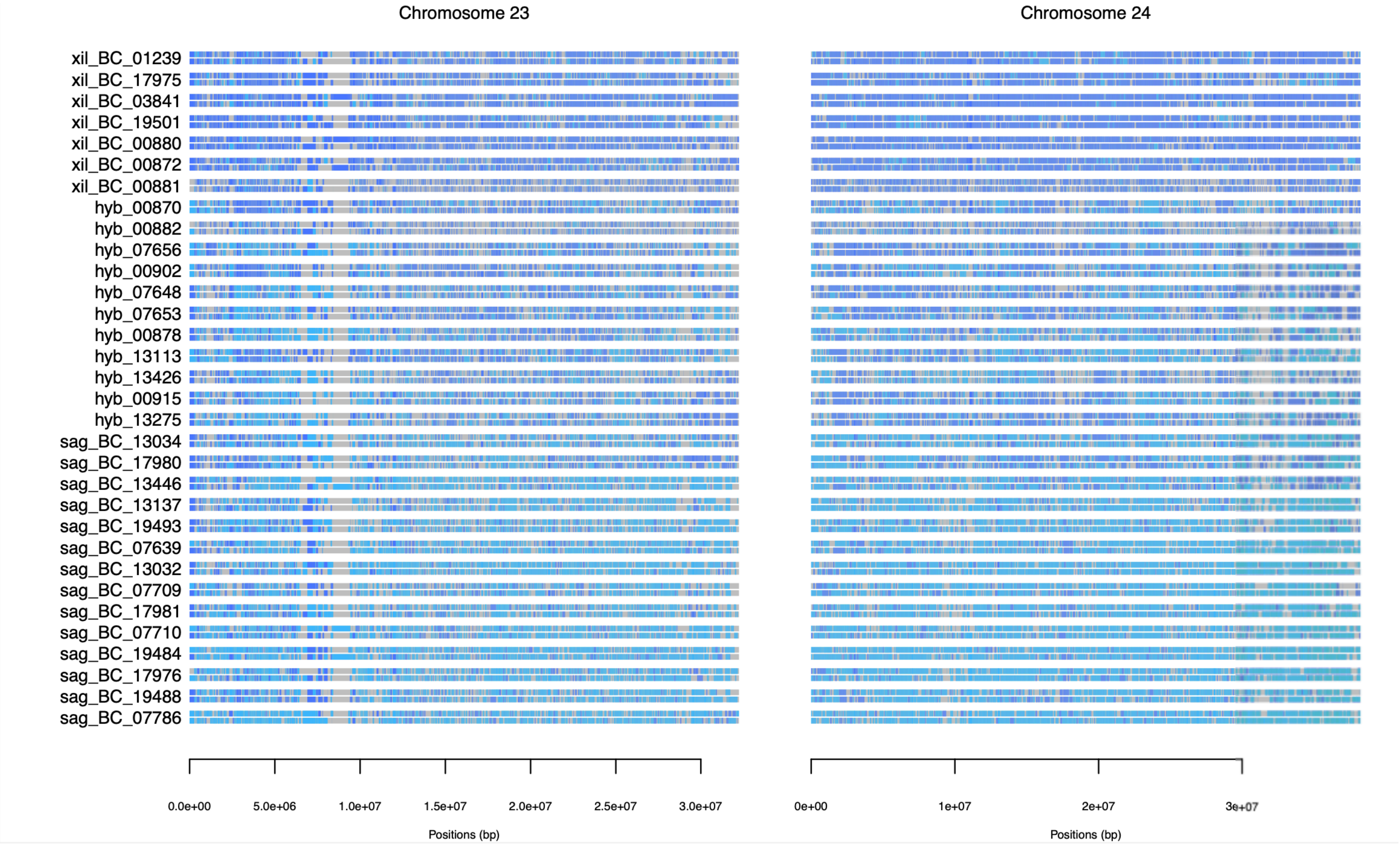

Supplementary Fig. 11 (continuation): Estimate of ancestry with chromopainter for hybrid individuals + backrosses across their 24 chromosomes inferred as *A. sagittae* (light blue) or *A. xiloaensis* (dark blue) with probability >0.8. Gray represents uncertain ancestry (probability <0.8). Source data are provided in Source Data file 2.

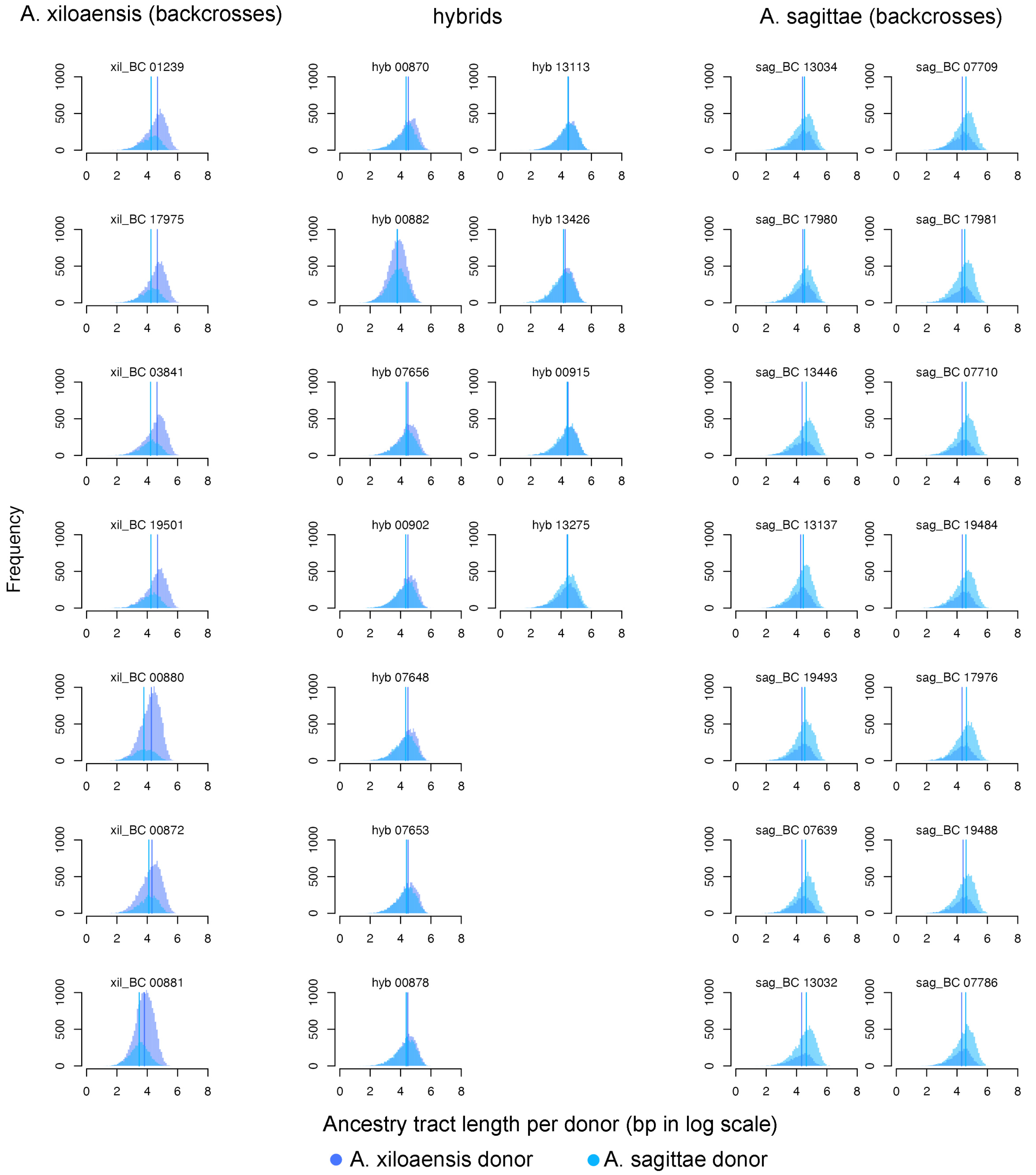

Supplementary Fig. 12: Histograms of ancestry tract length (bp) per individual and separated per donor (in log scale). Vertical lines indicate positions of medians. Source data is provided in Source Data file 2.

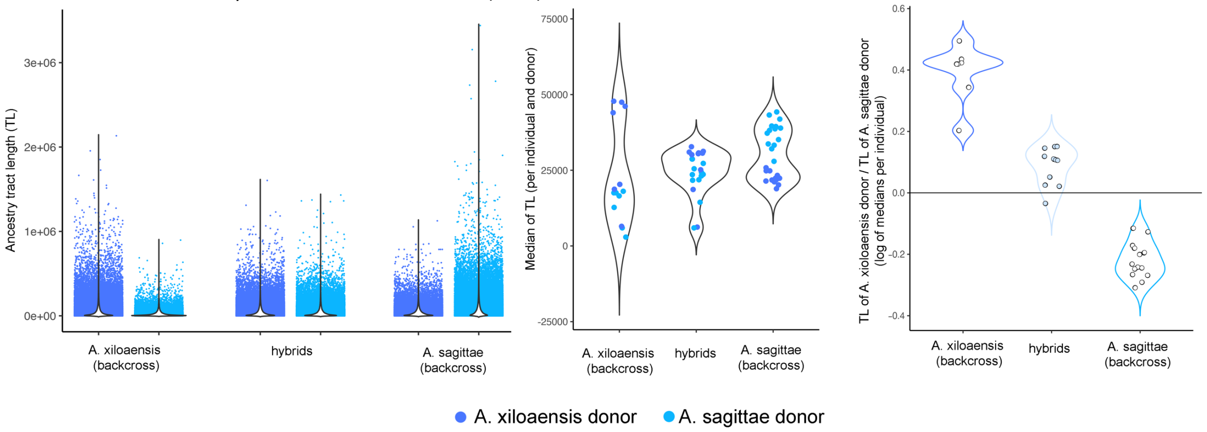

Supplementary Fig. 13: Violin plots showing: left, acestry tract length (TL) for backcrosses and hybrids and their donors; center, medians of donor TL obtained per individual for bacrosses and hybrids; right, donor ratio of TL medians for backcrosses and hybrids. In the later, horizontal line is indicating log ratio = 0, i.e. equal medians for both donors. Source data are provided as Source Data file 1 and 2.

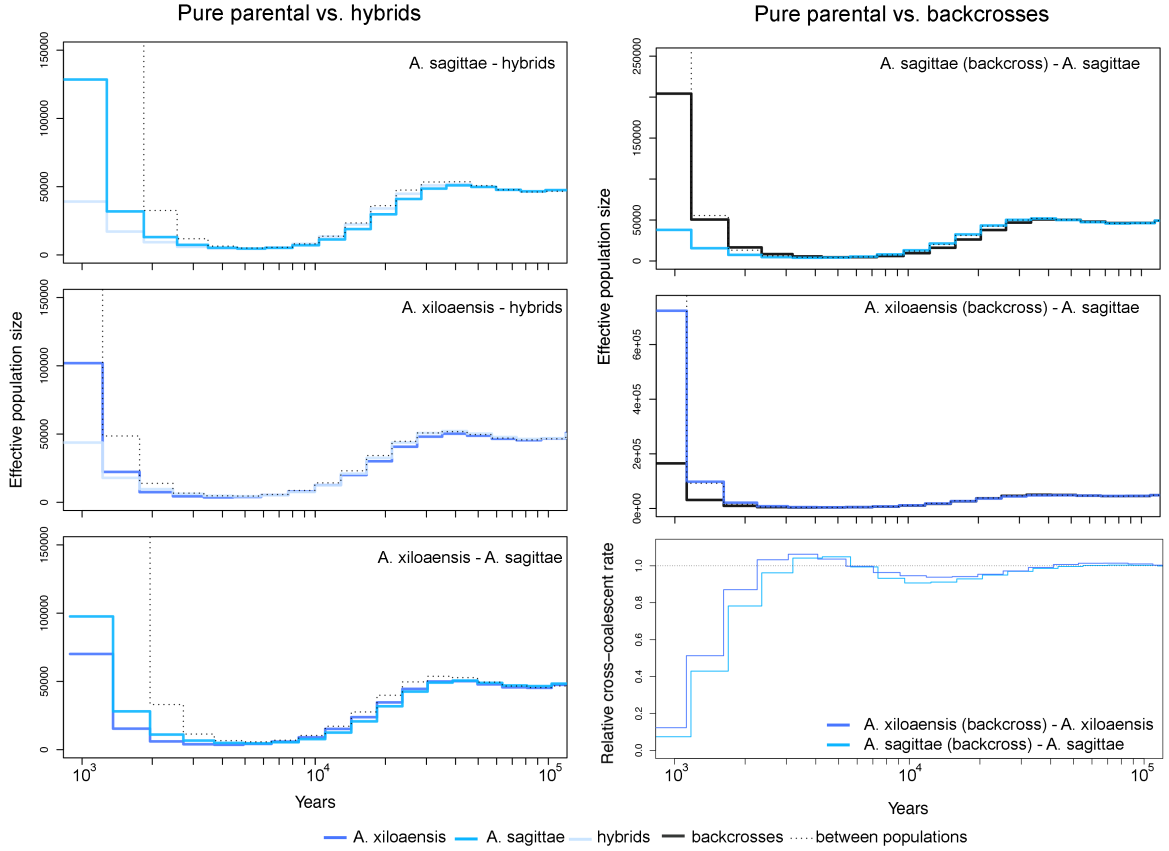

Supplementary Fig. 14: Left, changes in effective population size through time estimated using MSMC comparing hybrids and pure parental species. Right, changes in effective population size through time and relative cross coalescence rates estimated using MSMC comparing backcrosses and pure parental species. Source data are provided as Source Data file 1.

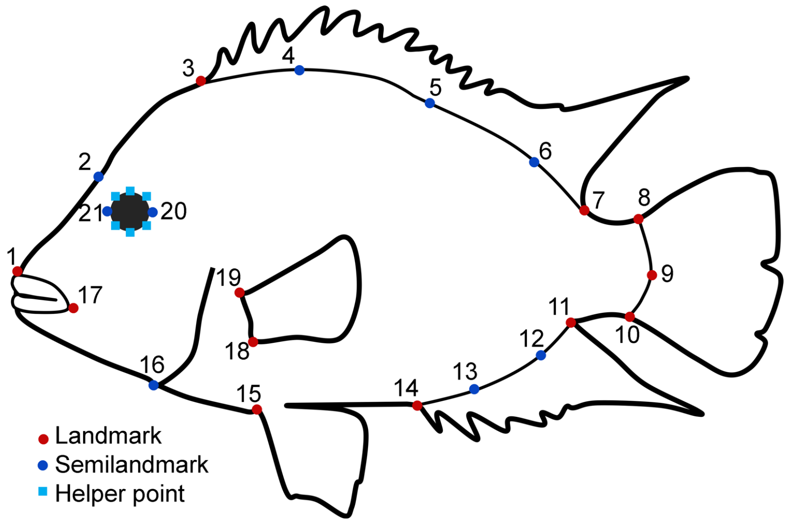

Supplementary Fig. 15: Landmark, semilandmark and helper point positions for geometric morphometric analyses. Raw Source Data is available in tps format in Source Data file 2.

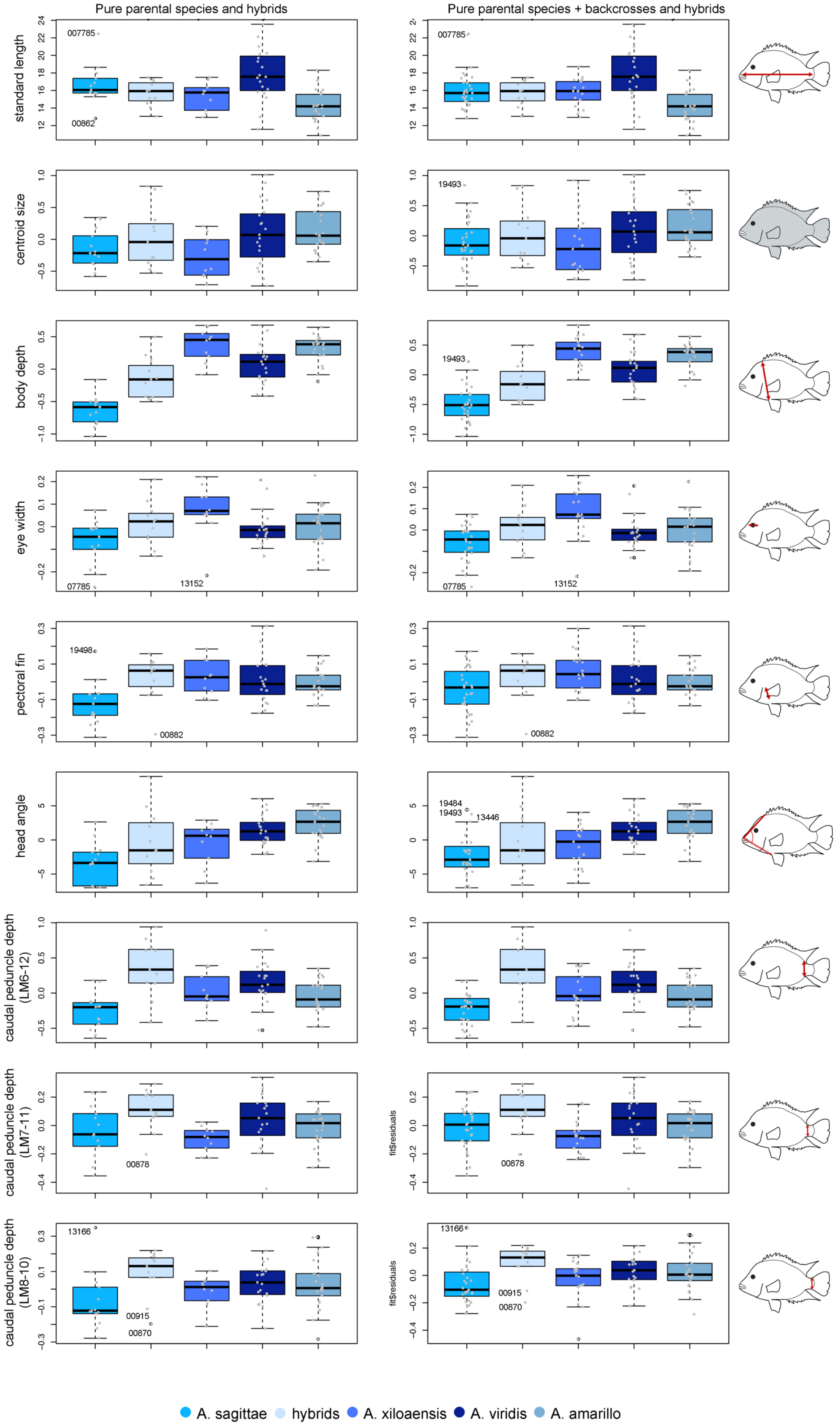

Supplementary Fig. 16: Box plots of different linear measures of all Midas cichlid species in CL Xiloá, displaying 5% and 95% quartile (lower and upper whiskers limits, respectively), 25% and 75% quantile (lower and upper box bounds, respectively) and 50% quantil in centre. For comparision, only pure parental and hybrid samples were included in the plots on the left, and backcrosses were classified as parental species in plots on the right. From the sencond row to the bottom, residuals were obtained after regressing variables with standard length (i.e., size correction). A total of biological independent *A. xiloaensis* n =10, *A. sagittae* n = 13, hybrids n = 11, backcrosses n = 21, *A. viridis* n = 19, *A. amarillo* n=21 IDs of outliers for the two parental species and hybrids are shown. Source data are provided as Source Data file 1.

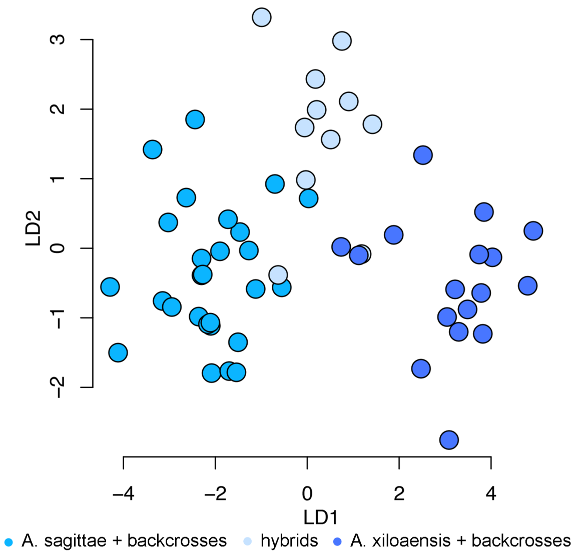

Supplementary Fig. 17: Scatter plot of LD1 and LD2 axes obtained from a linear discriminant function based on the geometric morphometric data and including all ingroup samples (pure parental species plus backcrosses and hybrids). Source data are provided as Source Data file 1.

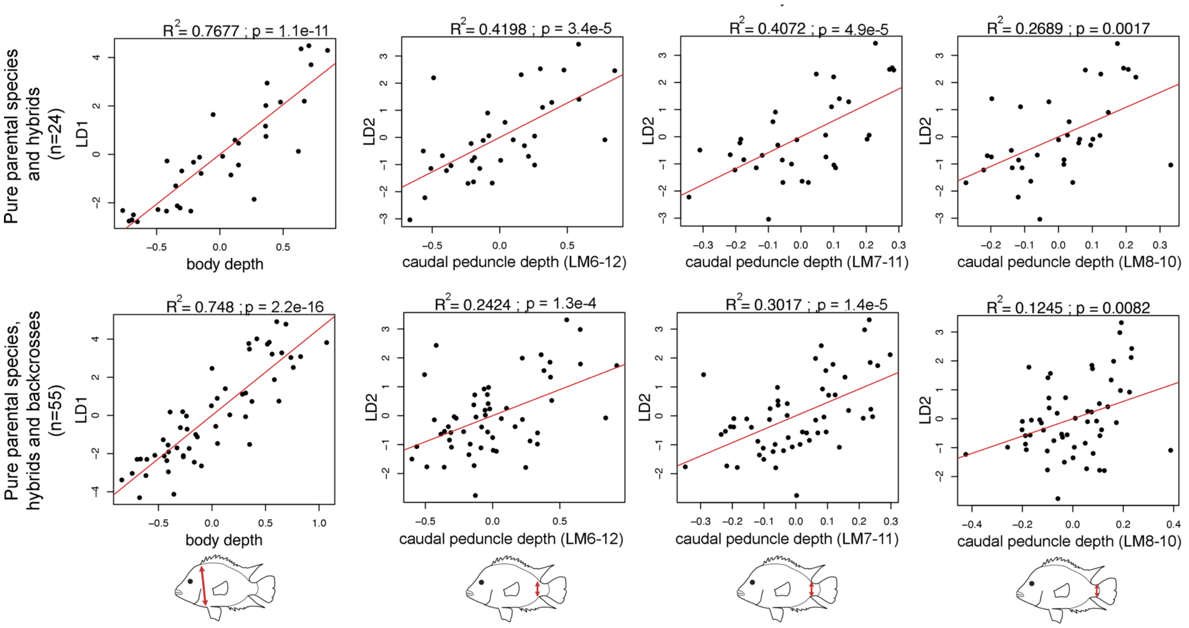

Supplementary Fig. 18: Linear correlations and two sided F-test result between LD1 (from dicriminant analysis, Supplementary Fig. 6) and body depth (left), as well as LD2 and caudal pendunce depth (using different LMs), considering only pure *A. sagittae*, *A. xiloaensis,* and hybrids (above) and all samples including backcrosses (below). All linear variables described in the materials and methods section were fitted (shown in Supplementary Fig. 15), but only significant correlations are shown here. Source data are provided as Source Data file 1.

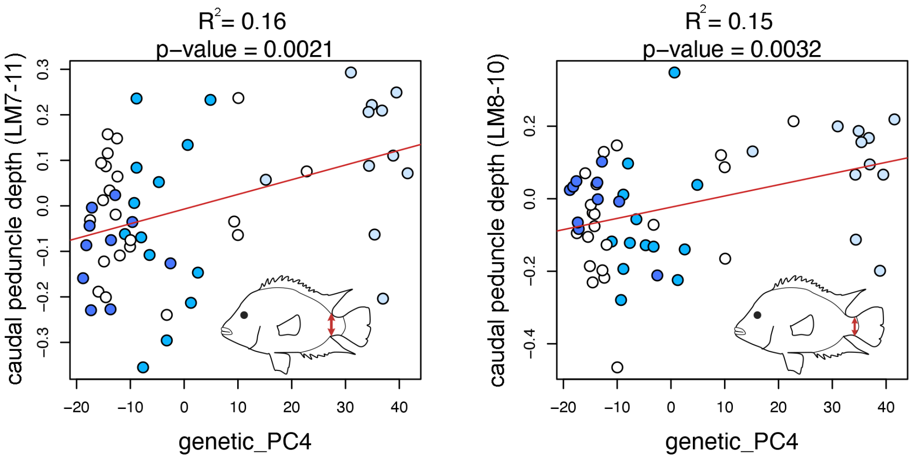

Supplementary Fig. 19: Scatter plots and linear models fit between the caudal peduncle depth (using linear distances between different LM sets (LM7-11 and 8-10); after correcting by standard length) and the genetic PC4. Statistical two sided F-test results: (left) F = 10.46 on 1 and 53 DF, p-value = 0.002105**; (right) F = 9.55 on 1 and 53 DF, p-value = 0.003184**. Source data are provided as Source Data file 1.

Supplementary Table 1. HyDe result (hybrid detection test, Z-test) based on 88,369 SNPs (sampled every 10 kb along the genome). Samples of *A. citrinellus* from Great Lake Managua were used as outgroup. Source data is available in Source Data file 2.

P1	Hybrid	P2	p-value	Z score	Gamma
A. xiloaensis	**hybrids**	***A. sagittae***	**0.00560****	**2.5362**	**0.628**
A. xiloaensis	hybrids	*A. viridis*	0.42332	0.3360305	0.966
A. xiloaensis	hybrids	*A. amarillo*	-0.58179	0.71964605	1.054
A. sagittae	hybrids	*A. viridis*	-1.42025	0.9222339	1.244
A. sagittae	hybrids	*A. amarillo*	-0.22096	0.58744054	1.022
A. viridis	hybrids	*A. amarillo*	1.41714	0.07821995	0.603

Supplementary Table 2: Private SNP counts per species (frequency cutoff = 0.5). A total of 3,745,928 SNPs were considered among 74 individuals in total: 21 *A. amarillo*, 11 hybrids, 19 *A. viridis*, 10 pure *A. xiloaensis* and 13 pure *A. sagittae*. Source data is provided in vcf format in Source Data file 2.

Chromosome	*A. amarillo*	hybrids	*A. viridis*	*A. xiloaensis*	*A. sagittae*
1	29	5	0	0	1
2	0	0	0	0	0
3	3	0	0	0	1
4	5	7	1	5	0
5	3	0	0	0	0
6	47	0	2	0	0
7	6	0	4	1	0
8	7	0	0	3	0
9	2	0	0	0	0
10	0	2	0	0	0
11	8	0	0	4	0
12	7	1	0	1	0
13	0	0	0	0	0
14	5	0	3	0	0
15	7	0	1	0	0
16	1	2	4	0	0
17	4	1	0	1	0
18	0	18	0	0	0
19	0	0	1	1	0
20	0	2	0	1	0
21	4	0	0	2	0
22	1	0	2	0	0
23	15	0	6	0	0
24	33	0	0	1	0
Total	187	38	24	20	2

Supplementary Table 3: Thirty-eight private SNPs found in eleven hybrids (frequency >= 0.5), their annotations, distance to the annotated gene, and information (using all Midas cichlids in CL Xiloá, n=95) on per-site SNP quality (QUAL), depth per site summed across all individuals (SUM_DEPTH and SUMSQ_DEPTH), and the mean and variation in depth per site averaged across all individuals (MEAN_DEPTH and VAR_DEPTH). These same statistics are shown in reference to all SNPs across the genome in Supplementary Figure 1. Source data are provided in vcf format in Source Data file 2.

CHR	Position	Type	Annotation	Distance (bp)	QUAL	SUM_DEPTH	SUMSQ_DEPTH	MEAN_DEPTH	VAR_DEPTH
1	30273478	intergenic region	Very long chain 3-oxoacyl-CoA reductase-B	59948	3697.28	1788.00	37262.00	18.82	38.40
1	30274360	intergenic region	Very long chain 3-oxoacyl-CoA reductase-B	60830	38670.20	1809.00	37607.00	19.04	33.62
1	30276999	intergenic region	Very long chain 3-oxoacyl-CoA reductase-B	63469	35817.20	1746.00	35808.00	18.38	39.56
1	30279393	intergenic region	Very long chain 3-oxoacyl-CoA reductase-B	65863	10340.30	1478.00	26950.00	15.56	42.08
1	30280072	intergenic region	Very long chain 3-oxoacyl-CoA reductase-B	66542	7216.31	1297.00	21057.00	13.65	35.63
4	23365900	missense variant	WW domain-binding protein 2	NA	3297.67	2027.00	47559.00	21.34	45.84
4	28266290	intergenic region	NA	17692	4080.26	1647.00	33419.00	17.34	51.76
4	28326507	downstream gene variant	Protein SCO1 homolog, mitochondrial	600	2974.47	1875.00	39841.00	19.74	30.15
4	28385217	intergenic region	Growth arrest-specific protein 7	28157	5120.13	2133.00	51589.00	22.45	39.34
4	28400388	intergenic region	Growth arrest-specific protein 7	12986	6565.44	1966.00	43950.00	20.69	34.73
4	28414990	intron variant	Growth arrest-specific protein 7	1493	7289.90	1814.00	38462.00	19.09	40.68
4	28523641	5 prime UTR variant	Dual specificity mitogen-activated protein kinase kinase 4	6510	15316.10	1980.00	44790.00	20.84	37.47
10	27466499	downstream gene variant	55 kDa erythrocyte membrane protein	2384	3855.22	1935.00	43035.00	20.37	38.53
10	27581197	intergenic region	Gamma-aminobutyric acid receptor subunit beta-4	9040	3664.46	2016.00	45734.00	21.22	31.41
12	1133713	intergenic region	Serologically defined colon cancer antigen 3 homolog	10389	4073.07	2009.00	47189.00	21.15	50.04
16	35299454	intergenic region	Poly [ADP-ribose] polymerase 4	59747	3800.12	1973.00	43387.00	20.77	25.65
16	35405550	intergenic region	Titin	163988	3607.78	1789.00	36347.00	18.83	28.27
17	14118258	intergenic region	mRNA-decapping enzyme 1B	21701	865.17	969.00	11637.00	10.20	18.65
18	12232293	intergenic region	RNA-binding Raly-like protein	9795	4100.28	2060.00	47696.00	21.68	32.20
18	12268414	intron variant	RNA-binding Raly-like protein	1898	4901.34	1923.00	42543.00	20.24	38.48
18	12271855	intron variant	RNA-binding Raly-like protein	5339	6497.46	1947.00	45229.00	20.49	56.66
18	12272707	intron variant	RNA-binding Raly-like protein	6191	169782.00	1519.00	27165.00	15.99	30.61
18	12272820	intron variant	RNA-binding Raly-like protein	6304	4628.54	1716.00	33980.00	18.06	31.74
18	12273625	intron variant	RNA-binding Raly-like protein	7109	6026.96	2008.00	45446.00	21.14	31.95
18	12273685	intron variant	RNA-binding Raly-like protein	7169	6397.05	2072.00	48494.00	21.81	35.13
18	12274867	intron variant	RNA-binding Raly-like protein	8351	27570.70	2124.00	50992.00	22.36	37.27
18	12275000	intron variant	RNA-binding Raly-like protein	8484	26925.20	2026.00	46646.00	21.33	36.58
18	12275314	intron variant	RNA-binding Raly-like protein	8798	24278.70	1913.00	41243.00	20.14	28.95
18	12275436	intron variant	RNA-binding Raly-like protein	8920	6629.32	2115.00	50377.00	22.26	35.00
18	12275475	intron variant	RNA-binding Raly-like protein	8959	24962.80	2079.00	48691.00	21.88	33.98
18	12276063	intron variant	RNA-binding Raly-like protein	9547	5616.11	1791.00	37223.00	18.85	36.79
18	12276106	intron variant	RNA-binding Raly-like protein	9590	18370.10	1949.00	42993.00	20.52	32.00
18	12276671	intron variant	RNA-binding Raly-like protein	10155	25102.60	2007.00	45445.00	21.13	32.39
18	12276760	intron variant	RNA-binding Raly-like protein	10244	6213.81	1981.00	44061.00	20.85	29.28
18	12281200	intron variant	RNA-binding Raly-like protein	302	6512.47	1634.00	31556.00	17.20	36.71
18	12334068	3 prime UTR variant	Leucine-rich repeat and coiled-coil domain-containing protein 1	188	5264.95	1983.00	44141.00	20.87	29.24
20	13306510	intron variant	Cadherin-like protein 26	2602	5296.93	1766.00	36868.00	18.59	42.97
20	13310654	intron variant	Cadherin-like protein 26	6746	5194.84	1813.00	39695.00	19.08	54.21

Supplementary Table 4. Result of gene ontology (GO) enrichment analysis (for the 20 most statistically significant results), for private alleles in hybrids. Annotations used as input are shown in Supplementary Table 3.

Enrichment FDR	N Genes	Pathway Genes	Fold Enrichment	Pathway	Genes
0.029	1	8	390.86	Respiratory chain complex IV assembly	sco1
0.029	2	247	25.32	Pos. reg. of metabolic proc.	dcp1a wbp2
0.029	1	4	781.72	Reg. of gene expression, epigenetic	wbp2
0.029	1	5	625.38	Copper ion homeostasis	sco1
0.029	1	8	390.86	Nuclear-transcribed mRNA catabolic process, deadenylation-dependent decay	dcp1a
0.029	1	5	625.38	Deadenylation-dependent decapping of nuclear-transcribed mRNA	dcp1a
0.029	1	7	446.70	RNA decapping	dcp1a
0.029	1	7	446.70	Methylguanosine-cap decapping	dcp1a
0.029	1	3	1042.30	Cellular copper ion homeostasis	sco1
0.031	1	10	312.69	RNA destabilization	dcp1a
0.031	1	10	312.69	MRNA destabilization	dcp1a
0.031	1	12	260.57	Cytochrome complex assembly	sco1
0.031	1	11	284.26	Pos. reg. of mRNA catabolic proc.	dcp1a
0.034	1	14	223.35	Cellular response to estrogen stimulus	wbp2
0.037	3	1305	7.19	Reg. of biosynthetic proc.	dcp1a lrrcc1 wbp2
0.037	3	1270	7.39	Reg. of nucleobase-containing compound metabolic proc.	dcp1a lrrcc1 wbp2
0.037	1	17	183.93	Response to estrogen	wbp2
0.037	1	23	135.95	Reg. of RNA stability	dcp1a
0.038	3	1415	6.63	Reg. of gene expression	dcp1a lrrcc1 wbp2
0.038	1	28	111.67	Neg. reg. of translation	dcp1a

Supplementary Table 5: Frequency of private alleles found in hybrids, compared to all other available Midas cichlid genomes taken from Kautt et al. (2020; Nature). Eight SNPs only found in hybrids and backcrosses are highlighted in grey. Total sampling = 453 individuals. Abbreviations: CHR: chromosome, POS: position, N: Number of individuals samples per species/group shown on right row, xil: *A. xiloaensis*, BC_xil: *A. xiloaensis* backrosses, hyb: hybrids, BC_sag: *A.* sagittae backcross, sag: *A. sagittae*, ama: *A. amarillo*, vir: *A. viridis*, cit: *A. citrinellus*, lab: *A. labiatus*, ast: *A. astorqui*, cha: *A. chancho*, fla: *A. flaveolus*, glo: *A. globosus*, sup: *A. supercilius*, zal: *A.* zaliousus, tol: *A. tolteca*. Source Data available in Source Data file 1.

	Lake	CL Xiloá							Managua		Nicaragua		CL Apoyo						CL As Leon	CL As Managua	CL Apoyeque	CL Masaya	Tiscapa
	Species	xil	BC_xil	hyb	BC_sag	sag	ama	vir	cit	lab	cit	lab	ast	cha	fla	glo	sup	zal	cit	tol	cit	cit	cit
CHR	POS/N	10	7	11	14	13	21	19	25	25	24	24	23	23	17	26	10	21	20	20	40	40	20
1	30273478	-	0.14	0.59	0.04	-	-	-	-	-	-	-	-	-	-	-	-	-	-	-	-	-	-
	30274360	-	0.14	0.59	0.04	-	-	-	0.18	0.10	0.04	0.23	0.43	0.28	0.06	0.06	0.25	0.02	0.63	-	-	0.10	0.03
	30276999	-	0.07	0.50	0.04	-	-	-	0.18	0.12	0.10	0.21	0.48	0.28	0.12	0.10	0.25	0.07	0.63	-	-	0.10	0.03
	30279393	-	0.14	0.55	0.04	-	-	-	0.06	0.02	0.04	0.15	-	0.04	-	-	-	-	-	-	-	0.10	-
	30280072	-	0.14	0.50	0.04	-	-	-	0.08	0.02	0.04	0.15	-	-	-	-	-	-	-	-	-	0.10	-
4	23365900	-	0.07	0.55	-	-	-	-	0.02	-	-	-	-	-	-	-	-	-	-	-	-	-	-
	28266290	-	0.07	0.59	0.04	-	-	-	-	-	-	-	-	-	-	-	-	-	-	-	-	-	-
	28326507	-	0.07	0.59	0.04	-	-	-	0.04	-	0.04	0.04	0.02	-	0.03	0.04	-	-	-	-	-	0.03	-
	28385217	-	0.14	0.59	0.04	-	-	-	-	-	-	-	-	-	-	-	-	-	-	-	-	-	-
	28400388	-	0.14	0.64	0.04	-	-	-	0.02	-	-	0.02	-	-	-	-	-	-	-	0.10	-	-	-
	28414990	-	0.14	0.59	0.04	-	-	-	-	0.06	-	-	-	-	-	-	-	-	-	0.05	-	0.19	-
	28523641	-	0.14	0.68	0.04	-	-	-	0.06	0.08	0.02	0.02	0.04	0.02	0.15	0.19	-	-	-	-	-	0.11	-
10	27466499	-	-	0.59	0.04	-	-	-	-	-	-	-	-	-	-	-	-	-	-	-	-	-	-
	27581197	-	-	0.55	-	-	-	-	-	-	-	-	-	-	-	-	-	-	-	-	-	-	-
12	1133713	-	0.07	0.50	0.18	-	-	-	-	0.02	-	-	-	-	-	-	-	-	-	0.03	-	-	-
16	35299454	-	0.07	0.55	-	-	-	-	-	-	-	-	-	-	-	-	-	-	-	-	-	-	-
	35405550	-	0.07	0.55	-	-	-	-	0.02	-	0.02	-	-	-	-	-	-	-	-	-	-	-	-
17	14118258	-	-	0.50	0.07	-	-	-	-	-	-	-	-	0.02	-	-	-	-	-	-	-	-	-
18	12232293	-	-	0.55	0.04	-	-	-	-	-	-	-	-	-	-	-	-	-	-	-	-	-	-
	12268414	-	-	0.55	0.04	-	-	-	0.04	-	0.06	0.08	-	-	-	-	-	-	-	-	0.01	-	-
	12271855	-	-	0.55	0.04	-	-	-	0.04	-	0.06	0.08	-	-	-	-	-	-	-	-	-	-	-
	12272707	-	-	0.55	0.07	-	-	-	0.04	-	0.06	0.08	-	-	-	-	-	-	-	-	-	-	-
	12272820	-	-	0.55	0.04	-	-	-	0.04	-	0.06	0.08	-	-	-	-	-	-	-	-	-	-	-
	12273625	-	-	0.50	0.04	-	-	-	0.04	-	0.04	0.08	-	-	-	-	-	-	-	-	-	-	-
	12273685	-	-	0.55	0.04	-	-	-	0.04	-	0.06	0.08	-	-	-	-	-	-	-	-	-	-	-
	12274867	-	-	0.55	0.04	-	-	-	0.04	-	0.08	0.13	-	-	-	-	-	-	-	-	-	0.01	-
	12275000	-	-	0.55	0.04	-	-	-	0.04	-	0.06	0.13	-	-	-	-	-	-	-	-	-	-	-
	12275314	-	-	0.55	0.04	-	-	-	0.04	-	0.08	0.15	-	-	-	-	-	-	-	-	-	-	-
	12275436	-	-	0.55	0.04	-	-	-	0.04	-	0.08	0.13	-	-	-	-	-	-	-	-	-	-	-
	12275475	-	-	0.55	0.04	-	-	-	0.04	-	0.08	0.13	-	-	-	-	-	-	-	-	-	-	-
	12276063	-	-	0.55	0.04	-	-	-	0.04	-	0.08	0.10	-	-	-	-	-	-	-	-	-	-	-
	12276106	-	-	0.55	0.04	-	-	-	0.04	-	0.08	0.10	0.02	-	-	-	-	-	-	-	-	-	-
	12276671	-	-	0.50	0.04	-	-	-	0.04	-	0.08	0.13	-	-	-	-	-	-	-	-	-	-	-
	12276760	-	-	0.55	0.04	-	-	-	0.04	-	0.08	0.13	-	-	-	-	-	-	-	-	-	-	-
	12281200	-	-	0.50	0.04	-	-	-	0.06	-	0.17	0.10	-	-	-	-	-	-	-	-	-	-	-
	12334068	-	-	0.55	0.18	-	-	-	-	-	-	-	-	-	-	-	-	-	-	-	-	-	-
20	13306510	-	-	0.50	0.14	-	-	-	0.02	-	-	-	-	-	-	-	-	-	-	0.03	0.01	0.04	-
	13310654	-	0.07	0.50	0.14	-	-	-	-	-	-	-	-	-	-	-	-	-	-	0.03	-	0.04	-

Supplementary Table 6. Summary statistics of inbreeding coefficent calculated along the genome (2,510,628 SNPs ± 31,789.87) on a per-individual basis using vcftools (F-statistic = 1 – observed heterozygosity / expected heterozygosity). Data source is available in Source Data file 1.

Group	Mean F-statistic ± s.d.
hybrids (n = 11)	-0.12268 ± 0.204
A. sagittae (n=13)	-0.06182 ± 0.073
A. sagittae + backcrosses (n = 27)	-0.0494 ± 0.064
A. xiloaensis (n=10)	0.0025 ± 0.054
A. xiloaensis + backcrosses (n = 17)	-0.0565 ± 0.158

Supplementary Table 7: Fifty-two diagnostic SNPs found in pure the parental species *A. xiloaensis* (n=10) and *A. sagittae* (n=13). Raw data is available in vcf format in Source Data file 2.

Chromosome	Position	Annotation
4	5969572	GTPase IMAP family member 8
4	5976617	GTPase IMAP family member 8
4	5977655	GTPase IMAP family member 8
4	5978898	GTPase IMAP family member 8
4	5980671	NA
4	5987109	NA
4	5998204	Band 3 anion exchange protein
4	5998485	Band 3 anion exchange protein
4	6000454	Band 3 anion exchange protein
4	6001793	Band 3 anion exchange protein
4	6019112	Ataxin-7-like protein 3
6	25501645	NA
8	11737616	NA
8	19406239	Tumor necrosis factor receptor superfamily member 16
8	19416369	Alpha-tectorin
11	27820225	CTP synthase 1
11	38567601	Activating signal cointegrator 1 complex subunit 3
11	38567605	Activating signal cointegrator 1 complex subunit 3
12	31358111	Semaphorin-6A
14	38750901	FRAS1-related extracellular matrix protein 2
14	38751714	FRAS1-related extracellular matrix protein 2
14	38751885	FRAS1-related extracellular matrix protein 2
14	38763047	Tripartite motif-containing protein 3
14	38983846	T-lymphoma invasion and metastasis-inducing protein 1
14	38986480	T-lymphoma invasion and metastasis-inducing protein 1
14	38996405	T-lymphoma invasion and metastasis-inducing protein 1
14	38997403	T-lymphoma invasion and metastasis-inducing protein 1
14	39003955	T-lymphoma invasion and metastasis-inducing protein 1
14	39004000	T-lymphoma invasion and metastasis-inducing protein 1
16	6809063	Tomoregulin-2
16	6810658	Tomoregulin-2
16	6810686	Tomoregulin-2
16	6812564	Tomoregulin-2
16	6814390	Tomoregulin-2
16	6814819	Tomoregulin-2
16	6818333	Tomoregulin-2
17	13684620	ELKS/Rab6-interacting/CAST family member 1
17	13687285	ELKS/Rab6-interacting/CAST family member 1
17	13690820	ELKS/Rab6-interacting/CAST family member 1
20	28125727	Serine/threonine-protein kinase mTOR
20	28135238	Agrin
20	28148990	Agrin
20	28151399	Agrin
24	23949788	Phospholipid-transporting ATPase IC
24	23961967	Phospholipid-transporting ATPase IC
24	24080633	Zinc finger and BTB domain-containing protein 7A
24	24098249	Transcription factor E2-alpha
24	32563684	Myomegalin
24	32609243	Myomegalin
24	32611777	Myomegalin
24	32618410	Myomegalin
24	32618606	Myomegalin

Supplementary Table 8: Result of gene ontology (GO) enrichment analysis (20 lowest enrichement FDR), for annotations in fixed SNPs in parental species. Annotation used as input are listed in Supplementary Fig. 3.

Enrichment FDR	nGenes	Pathway Genes	Fold Enrichment	Pathway	Genes
0.101	1	4	390.86	Histone deubiquitination	ATXN7L3
0.101	1	5	312.69	Reg. of myelination	mtor
0.101	1	3	521.15	Pos. reg. of myelination	mtor
0.101	1	5	312.69	Reg. of nervous system proc.	mtor
0.101	1	3	521.15	Otolith tethering	tecta
0.101	1	4	390.86	Activation of GTPase activity	tiam1b
0.104	1	6	260.57	Receptor clustering	agrn
0.104	1	9	173.72	Skin development	mtor
0.104	5	1977	3.95	System development	slc4a1a mtor agrn tecta ctps1b
0.104	1	11	142.13	Acetylcholine receptor signaling pathway	agrn
0.104	1	11	142.13	Postsynaptic signal transduction	agrn
0.104	1	11	142.13	Response to acetylcholine	agrn
0.104	1	11	142.13	Cellular response to acetylcholine	agrn
0.104	1	11	142.13	G protein-coupled acetylcholine receptor signaling pathway	agrn
0.104	1	17	91.97	Pyrimidine nucleoside triphosphate metabolic proc.	ctps1b
0.104	1	15	104.23	Pyrimidine ribonucleoside triphosphate biosynthetic proc.	ctps1b
0.104	1	15	104.23	CTP metabolic proc.	ctps1b
0.104	2	246	12.71	Nervous system proc.	mtor tecta
0.104	1	15	104.23	CTP biosynthetic proc.	ctps1b
0.104	1	17	91.97	Glutamine metabolic proc.	ctps1b

Supplementary Table 9: Linear model (two sided F-test) results for ancestry tract length comparisons of hybrids and backcrosses, based on medians obtained per individual and their donors. Model: median ancestry tract lengths ~ group*donor; F = 12.76 on 5 and 58 DF, p-value = 2.24 x10^{-8}***. Source Data is available in Source Date file 1.

	Estimate Std.	Error	t value	p-value
(Intercept)	21852	2296	9.518	1.8 x10^{-13} ***
A. sagittae (backcrosses)	15568	3068	5.074	4.3 x10^{-6} ***
A. xiloaensis (backcrosses)	-8786	3682	-2.386	0.02030*
A. xiloaensis donor	5056	3247	1.557	0.12490
A. sagittae (backcrosses): A. xiloaensis donor	-20042	4339	-4.619	2.1x10^{-5} ***
A. xiloaensis (backcrosses): A. xiloaensis donor	14887	5207	2.859	0.00589**
Residual standard error: 7615 on 58 degrees of freedom				
Multiple R-squared: 0.5239, Adjusted R-squared: 0.4828				
F-statistic: 12.76 on 5 and 58 DF, p-value 1.24 x10^{-10}				

Supplementary Table 10: Two-sided t-test results for standard length (SL) comparisons among hybrids and the two parental species. Source Data is available in Source Data file 1.

Excluding backcrosses	n	t-value	p-value
A. sagittae	13	1.367	0.181
A. xiloaensis	10	-0.403	0.689
Model df = 31; p = 0.1912; F-statistic = 1.74			
Including backcrosses			
A. sagittae + backcrosses	27	0.415	0.68
A. xiloaensis + backcrosses	17	0.136	0.892
Model df = 52; p = 0.9028; F-statistic = 0.1025			

Supplementary Table 11: Linear model (two sided F-test) results for caudal depth (LM6-12, SL corrected) comparisons among hybrids and the two parental species and backcrosses.

Excluding backcrosses	n	t-value	p-value
A. sagittae	13	-4.602	7.15 x10^{-5}***
A. xiloaensis	10	-2.437	0.021*
SL co-variable		10.174	3.1 x10^{-11}***
Model df = 30; p = 2.39 x10^{-10} ***; F-statistic = 38.05			
Pure parental + backcrosses			
A. sagittae + backcrosses	27	-5.776	4.6 x10^{-7}***
A. xiloaensis + backcrosses	17	-3.132	0.00288**
SL co-variable		13.222	2.2 x10^{-16} ***
Model df = 51; p = 2.2 x10^{-16} ***; F-statistic = 66.79			
All as separate groups			
A. sagittae (backcrosses)	14	-4.934	9.7 x10^{-7}***
A. xiloaensis (backcrosses)	7	-2.419	0.0193*
A. sagittae	13	-4.9334	9.7 x10^{-7}***
A. xiloaensis	10	-2.733	0.0087**
SL co-variable		12.220	2.2 x10^{-16}***
Model df = 49; p = 2.2 x10^{-16} ***; F-statistic = 38.53			
