# Supplementary material for: Early stages of sympatric homoploid hybrid speciation in crater lake cichlid fishes
Source: Nat Commun. 2022 Oct 6;13:5893. doi: 10.1038/s41467-022-33319-4 (PMC9537415; doi:10.1038/s41467-022-33319-4)
Supplement: Supplementary file 2 — Reporting Summary [file 41467_2022_33319_MOESM2_ESM.pdf]

nature portfolio

Corresponding author(s): Prof. Axel Meyer

Last updated by author(s): Sep 1, 2022

Reporting Summary

Nature Portfolio wishes to improve the reproducibility of the work that we publish. This form provides structure for consistency and transparency in reporting. For further information on Nature Portfolio policies, see our Editorial Policies and the Editorial Policy Checklist.

Statistics

For all statistical analyses, confirm that the following items are present in the figure legend, table legend, main text, or Methods section.

n/a	Confirmed	
☐	☒	The exact sample size (*n*) for each experimental group/condition, given as a discrete number and unit of measurement
☐	☒	A statement on whether measurements were taken from distinct samples or whether the same sample was measured repeatedly
☐	☒	The statistical test(s) used AND whether they are one- or two-sided<br>*Only common tests should be described solely by name; describe more complex techniques in the Methods section.*
☒	☐	A description of all covariates tested
☒	☐	A description of any assumptions or corrections, such as tests of normality and adjustment for multiple comparisons
☐	☒	A full description of the statistical parameters including central tendency (e.g. means) or other basic estimates (e.g. regression coefficient) AND variation (e.g. standard deviation) or associated estimates of uncertainty (e.g. confidence intervals)
☐	☒	For null hypothesis testing, the test statistic (e.g. *F*, *t*, *r*) with confidence intervals, effect sizes, degrees of freedom and *P* value noted<br>*Give P values as exact values whenever suitable.*
☐	☒	For Bayesian analysis, information on the choice of priors and Markov chain Monte Carlo settings
☒	☐	For hierarchical and complex designs, identification of the appropriate level for tests and full reporting of outcomes
☒	☐	Estimates of effect sizes (e.g. Cohen's *d*, Pearson's *r*), indicating how they were calculated

Our web collection on statistics for biologists contains articles on many of the points above.

Software and code

Policy information about availability of computer code

Data collection	Geometric morphometric landmark data acquisition was performed with tpsDig v2.32
Data analysis	Genomics: freebayes v1.1.0, BWA mem v0.7.15, vcflib in vcffilter v1.0.4, vcftools v0.1.15, plink v1.90/v2.00, SHAPEIT2 v.2.r900, adegenet v2.1.2, admixture v1.3, SVDquartets in PAUP* v4.0, introgress v1.2.3, HIest v1.0, HyDe v1.0, MSMC v2.1.2, MSci in bpp v4.4<br>Morphometrics: tpsDig v2.32, R v3.6.1, geomorph v3.0.6, MASS v7.3.<br>Stable isotopes analyses: R v3.6.1

For manuscripts utilizing custom algorithms or software that are central to the research but not yet described in published literature, software must be made available to editors and reviewers. We strongly encourage code deposition in a community repository (e.g. GitHub). See the Nature Portfolio guidelines for submitting code & software for further information.

Data

Policy information about availability of data

All manuscripts must include a data availability statement. This statement should provide the following information, where applicable:

- Accession codes, unique identifiers, or web links for publicly available datasets
- A description of any restrictions on data availability
- For clinical datasets or third party data, please ensure that the statement adheres to our policy

Whole genome resequencing data of all 120 samples available in NCBI/SRA PRJEB38173 [https://www.ebi.ac.uk/ena/browser/view/PRJEB38173?show=reads],

reference genome available in DDBJ/ENA/GenBank under accesion JACBYM000000000 [https://www.ncbi.nlm.nih.gov/nuccore/JACBYM000000000.1]. Geometric morphometric and stable isotope data available in Figshare (https://figshare.com/projects/Early_stages_of_sympatric_homoploid_hybrid_speciation_in_crater_lake_cichlid_fishes/132428

Field-specific reporting

Please select the one below that is the best fit for your research. If you are not sure, read the appropriate sections before making your selection.

☐ Life sciences ☐ Behavioural & social sciences ☒ Ecological, evolutionary & environmental sciences

For a reference copy of the document with all sections, see nature.com/documents/nr-reporting-summary-flat.pdf

Ecological, evolutionary & environmental sciences study design

All studies must disclose on these points even when the disclosure is negative.

Study description	This study comprises whole-genome re-sequencing of 120 individuals geometric morphometrics of 95 individuals and stable isotope data of 59 individuals of Midas cichlids from Nicaragua. All data was collected from wild caught samples.
Research sample	This work is based on 120 re-sequenced genomes (Kautt et al. 2020; doi.org/10.1038/s41586-020-2845-0) and morphometric data from individuals. We aimed to sample at least 10 individuals per species whenever possible. Specifically, between 2003 and 2015 the following specimens were sampled in Crater Lake Xiloá: A. sagittae (n=13), A. xiloaensis (n=10), hybrids (n=11), backcrosses (n=21), and other Midas species in Crater Lake Xiloá, A. viridis (n=19), A. amarillo (n=21). In addition, the outgroup A. citrinellus (n=25) were sampled from Great Lake Managua. For stable isotope analyses (SIA), individuals were collected in different expeditions from 2010-2014 with 28 individuals taken from (Elmer et al. 2014; doi.org/10.1038/ncomms6168) plus one individual from Rometsch et al. (in prep.) and 30 new individuals. In total, we collected SIA information from a total of 9 individuals of hybrids, 10 A. sagittae, 11 A. xiloaensis, 19 A. viridis and 10 A. amarillo. Age could not be determined reliable in wild-caught fish, but all sampled individuals were, based on their size, adult fish.
Sampling strategy	Fish were collected with gill nets or by harpooning in Crater Lake Xiloá. Fish from the Great Lake Managua were obtained from local fisherman in Mateares. To obtain sufficient power (estimated based on previous studies in non-model organisms; the necessary sample size is impossible to asses in advance as it depends, for example, in the number of pure hybrids and backcrosses detected with genomic data, as they were unknown prior to this study) for the conducted analyses we aimed to sample around 10 individuals per species as the minimum number, whenever possible. All 95 individuals from Crater Lake Xiloa and 25 of A. critrinellus from Great Lake Managua included in Kautt et al. 2020; doi.org/10.1038/s41586-020-2845-0 were included here for genomic and morphometric analyses. To maximally reduce allometric effects only adult fishes were sampled. We included all stable isotope data of Elmer et al. 2014; doi.org/10.1038/ncomms6168 for which genomic data was available, in addition to one sample from Rometsch et al. (in prep.) and 30 new samples. For all individuals included in SIA, species identification was first assigned based on their morphology (photograph) and later confirmed based on genomic data, either included in this study (whole genome sequences) or from a previous study using RADseq data (Kautt et al. 2016; doi.org/10.1371/journal.pgen.1006157).
Data collection	Adult fishes were collected in field expeditions of the Meyer lab to Nicaragua between 2003 and 2015, by Axel Meyer and Andreas Kautt, plus other members of the Meyer lab. Photographs were taken in the field using a digital camera and used for geometric morphometric data generation, using 27 landmarks, semilandmarks and helper points for 95 ingroup species from Crater Lake Xiloá. Geometric morphometric data was collected by Melisa Olave. Stable isotope ratios (SIA) of carbon (δ13C) and nitrogen (δ15N) were calculated from white muscle tissue (taken from severed heads, dorso-posterior near the lateral line) in the Limnological Institute at the University of Konstanz. Individuals were analyzed by gas chromatography combustion isotope ratio mass spectrometry (GC-C-IRMS). New SIA data was collected by Melisa Olave.
Timing and spatial scale	Adult fish were collected in field expeditions of the Meyer lab to Nicaragua in 2003, 2007, 2010, 2013, 2014 and 2015. Crater Lake Xiloá was visited in different opportunities during these sampling years, until complete a representation of a minimum of 10 individuals per species (see also sampling strategy).
Data exclusions	No data were excluded from analyses
Reproducibility	Raw and intermediate genomic data are publicly available: ENA PRJEB38173 (https://www.ebi.ac.uk/ena/browser/view/PRJEB38173?show=reads), and the subset used in this study is available in Figshare (https://figshare.com/projects/Early_stages_of_sympatric_homoploid_hybrid_speciation_in_crater_lake_cichlid_fishes/132428). Morphometric and stable isotope data are available on Figshare (https://figshare.com/projects/Early_stages_of_sympatric_homoploid_hybrid_speciation_in_crater_lake_cichlid_fishes/132428). To test reproducibility and robustness we used a bootstrap replication for the species tree inference in SVDquartets (200 pseudo replicates), as well as 100 bootstrap pseudoreplicates for concordance factors calculations and 10 replicates for MSMC analysis. Confident intervals were obtained for our demographic model in MSci. Attempts at replication were successful.
Randomization	Individuals within species included in this study were sampled randomly. As all subsequent analyses were performed on all individuals, no randomization was necessary.
Blinding	Pure parental, hybrids and backcrosses were collected blind and their exact identification was only possible based on detailed genomic analysis. All morphometric and stable isotope data were collected blind to the identity of species.

Did the study involve field work? ☒ Yes ☐ No

Field work, collection and transport

Field conditions	Fishes were collected over a twelve year time period. Environmental conditions were not relevant (i.e. for sampling) and are therefore not reported here.
Location	Fish were collected from Crater Lake Xiloa (12º13'16''N 86º19'16''W) and Great Lake Managua (12º 14'10''N 86º25'48''W), Nicaragua
Access & import/export	All field work and export of samples was conducted only after approval by the local authorities, the Ministerio del Ambiente y los Recursos Naturales (MARENA), Nicaragua (permit numbers DGRNB-ACHL-0078. DRNB-IC-006-2007, No. 026-11007/DGAP, DGPN/DB-27-2010, DGPN-DB-004-2013, DGPN/DB-011-2014, DGPN/DB-IC-015-2015.
Disturbance	Midas cichlids are very abundant and a common food source in Nicaragua. The extractions of a limited number of animals for this study is therefore very unlikely to have caused any kind of disturbance of this natural system.

Reporting for specific materials, systems and methods

We require information from authors about some types of materials, experimental systems and methods used in many studies. Here, indicate whether each material, system or method listed is relevant to your study. If you are not sure if a list item applies to your research, read the appropriate section before selecting a response.

Materials & experimental systems

n/a	Involved in the study
☒	☐ Antibodies
☒	☐ Eukaryotic cell lines
☒	☐ Palaeontology and archaeology
☒	☐ Animals and other organisms
☒	☐ Human research participants
☒	☐ Clinical data
☒	☐ Dual use research of concern

Methods

n/a	Involved in the study
☒	☐ ChIP-seq
☒	☐ Flow cytometry
☒	☐ MRI-based neuroimaging
